# Acoustic Properties of Metal-Organic Frameworks

**DOI:** 10.34133/2021/9850151

**Published:** 2021-06-01

**Authors:** Zhi-Gang Li, Kai Li, Li-Yuan Dong, Tian-Meng Guo, Muhammad Azeem, Wei Li, Xian-He Bu

**Affiliations:** ^1^School of Materials Science and Engineering, Tianjin Key Lab of Metal and Molecule-Based Material Chemistry, Nankai University, Tianjin 300350, China; ^2^School of Physics and Wuhan National Laboratory for Optoelectronics, Huazhong University of Science and Technology, Wuhan 430074, China

## Abstract

Metal-organic frameworks (MOFs) have attracted significant attention in the past two decades due to their diverse physical properties and associated functionalities. Although numerous advances have been made, the acoustic properties of MOFs have attracted very little attention. Here, we systematically investigate the acoustic velocities and impedances of 19 prototypical MOFs *via* first-principle calculations. Our results demonstrate that these MOFs exhibit a wider range of acoustic velocities, higher anisotropy, and lower acoustic impedances than their inorganic counterparts, which are ascribed to their structural diversity and anisotropy, as well as low densities. In addition, the piezoelectric properties, which are intimately related to the acoustic properties, were calculated for 3 MOFs *via* density functional perturbation theory, which reveals that MOFs can exhibit significant piezoelectricity due to the ionic contribution. Our work provides a comprehensive study of the fundamental acoustic properties of MOFs, which could stimulate further interest in this new exciting field.

## 1. Introduction

Metal-organic frameworks (MOFs), constructed by metal ions/clusters and organic ligands *via* directional coordination bonds, have attracted wide attention during the past two decades since the homogeneous hybrid nature gives rise to the integration of properties from both the inorganic and organic components [1]. On the one hand, porous MOFs have shown promising application potential in gas storage and separation, catalysis, and drug delivery [2]; on the other hand, this emergent class of materials can exhibit diverse physical properties, including fluorescence, ferromagnetism, ferroelectricity, and multiferroicity [3–7]. Although there are numerous studies on the diverse properties and functionalities of MOFs [8], little attention has been paid to exploring an important area, namely, the acoustic properties [9]. Very recently, the application of acoustics and energy-transfer mechanisms in MOFs has been experimentally validated [10, 11], which prompts the necessity to systematically study the acoustic properties of MOFs. The most fundamental aspect of acoustics is to study the propagation of sound in materials, which includes the understanding of the acoustic velocity and acoustic impedance. Both of these parameters are closely related to the elastic properties of a given material and are determined by the stiffness constant and density [12]. Compared with purely inorganic (i.e., oxide) and organic (i.e., polymer) materials, MOFs have intermediate stiffness and density, which could enable them to show very different acoustic properties [13–15]. In this context, it is highly interesting to explore the fundamental acoustic velocity and acoustic impedance of MOFs and compare them with those of their purely inorganic and organic counterparts. In addition, it is well known that MOFs exhibit significant structural diversity and chemical variability; therefore, they are expected to show diverse acoustic properties, similar to their other properties [16]. Furthermore, the comprehensive study of the basic acoustic properties of MOFs is also of great importance for exploring their application potential in acoustics, such as acoustic switching devices and sensors.

In this work, we present a systematic study of the fundamental acoustic properties of 19 prototypical MOFs *via* first-principle calculations and compare them with those of traditional inorganic materials having similar structures. At the same time, potential acoustic applications of MOFs are proposed based on these properties. Moreover, the piezoelectric properties of several MOFs are calculated using density functional perturbation theory, and the advantageous properties of these MOFs over their inorganic counterparts are comprehensively discussed from the viewpoint of potential applications.

## 2. Results

### 2.1. Crystal Structure

**Figure 1:**
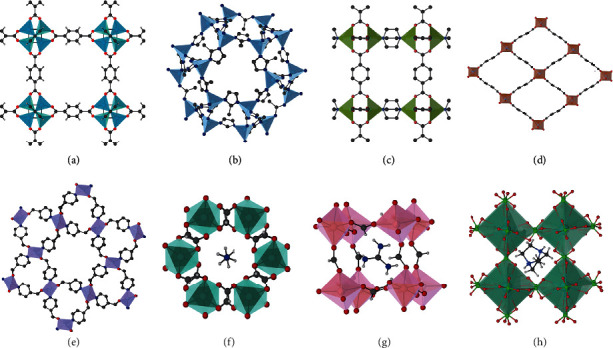
Crystal structures of some prototypical MOFs. From (a) to (h): MOF-5, ZIF-8, DMOF-1, MIL-53(Ga)lp, QMOF-1, NH_4_ZnH(COO)_3_, GuaZn(HCOO)_3_, and DABCOH_2_K(ClO_4_)_3_.

To study the acoustic properties of MOFs, we calculated the acoustic quantities of 22 materials, which included 19 prototypical MOFs and 3 inorganic materials for comparison. According to the chemical compositions and structural features, these 19 MOFs can be classified into 7 categories, including isoreticular MOF-5 [Zn_4_O(BDC)_3_, BDC = 1, 4 − benzendicarboxylate] [17]; ZIF-8 [Zn(mim)_2_, mim = 2 − methylimidazolate] and MAF-7 [Zn(mtz)_2_, mtz = 3 − methyl − 1, 2, 4 − triazolate] [18, 19]; DMOF-1sq and DMOF-1loz [Cu_2_(BDC)_2_(DABCO), DABCO = 1, 4 − diazabicyclo[2.2.2]octane] [20]; MIL-47 [V^IV^(O)(BDC)], MIL-53(Al)lp [Al(OH)(BDC)], and MIL-53(Ga)lp [Ga(OH)(BDC)] [21, 22]; QMOF-1 [Zn(ISN)_2_, ISN = isonicotinate] and QMOF-2 [InH(BDC)_2_] [23]; metal formate frameworks [AB(HCOO)_3_, where A = Na, MA (CH_3_NH_3_^+^), Gua (C(NH_2_)_3_^+^), DMA ((CH_3_)_2_NH_2_^+^), Hyz (NH_2_NH_3_^+^), NH_4_; B = Mn, Co, Zn, Mg] [24–30]; and a unique dense organic-inorganic framework, DABCOH_2_K(ClO_4_)_3_ [31]. In addition, the three traditional inorganic materials, quartz, BaTiO_3_, and sodalite [Na_4_Al_3_(SiO_4_)_3_], are selected for comparison with the structurally similar QMOFs, perovskite formates, and ZIF-8 and MAF-7, respectively. The structures of these materials are depicted in Figure 1.

### 2.2. Acoustic Velocity

Generally, there are two types of acoustic waves: pressure waves (p-waves, longitudinal waves) and shear waves (s-waves, transverse waves). Here, we divide the velocity of shear waves into the velocities of pure-s-waves (*v*_1_) and quasi-s-waves (*v*_2_). The quasi-s-waves have a small longitudinal wave component, which means that they are not purely transverse waves. The velocities of p-waves (*v*_3_) and s-waves are determined by the Young's moduli and shear moduli of the materials, respectively. As the Young's moduli of most materials are greater than their shear moduli, the magnitudes of these wave velocities have the following trend: *v*_1_ < *v*_2_ < *v*_3_.

The calculated independent elastic constants of 15 materials by us and 7 materials by others all conform to the elastic stability criterion and are tabulated in Table S1 along with some experimentally measured densities [32–34]. Meanwhile, the acoustic velocities were obtained by solving the Christoffel equations based on the elastic constants and densities. The 3D surfaces of the acoustic velocities for these 22 materials are presented in Figs. S1-S21.

**Figure 2:**
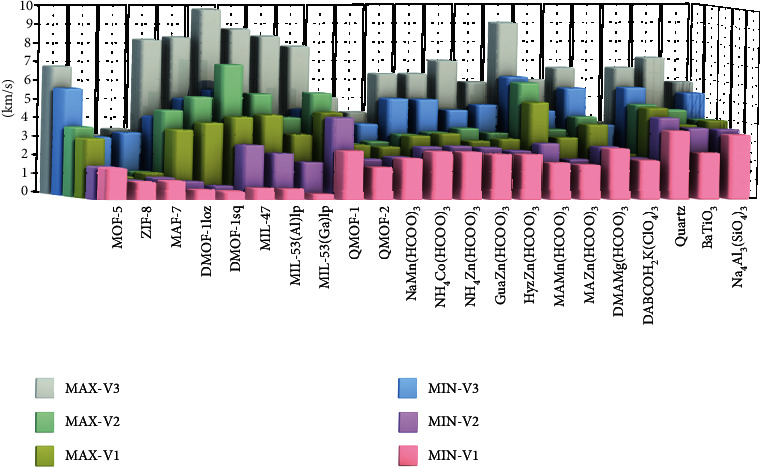
Maximum and minimum velocities of three types of acoustic waves propagating in the 22 materials.

Figure 2 shows the maximum and minimum values of *v*_1_, *v*_2_, and *v*_3_ in these 22 materials, which demonstrate that acoustic waves propagate in these MOF crystals with a wide range of velocities. For the maximum values of all three velocities, there are no obvious boundaries between the 19 MOFs and 3 inorganic materials. These results are reasonable considering the medium maximum moduli and low densities of MOFs. A similar trend is also found between the porous and dense MOFs. Notably, the maximum velocity values of some MOFs are even higher than those of quartz, BaTiO_3_,_,_ and sodalite. For example, the *v*_3_ value of MIL-47 is 9.94 km/s, which is 138% larger than that of BaTiO_3_. In terms of the minimum values of the three velocities, *v*_3min_ and *v*_2min_ are comparable with those of inorganic materials. However, the *v*_1min_ values of dense MOFs are comparable with those of inorganic materials and generally higher than those of porous MOFs. This is because the minimum shear moduli of some porous MOFs studied here are significantly lower than those of the dense systems and inorganic materials. For instance, MIL-53(Ga)lp has the lowest *v*_1min_ of 0.219 km/s among all 19 MOFs.

**Figure 3:**
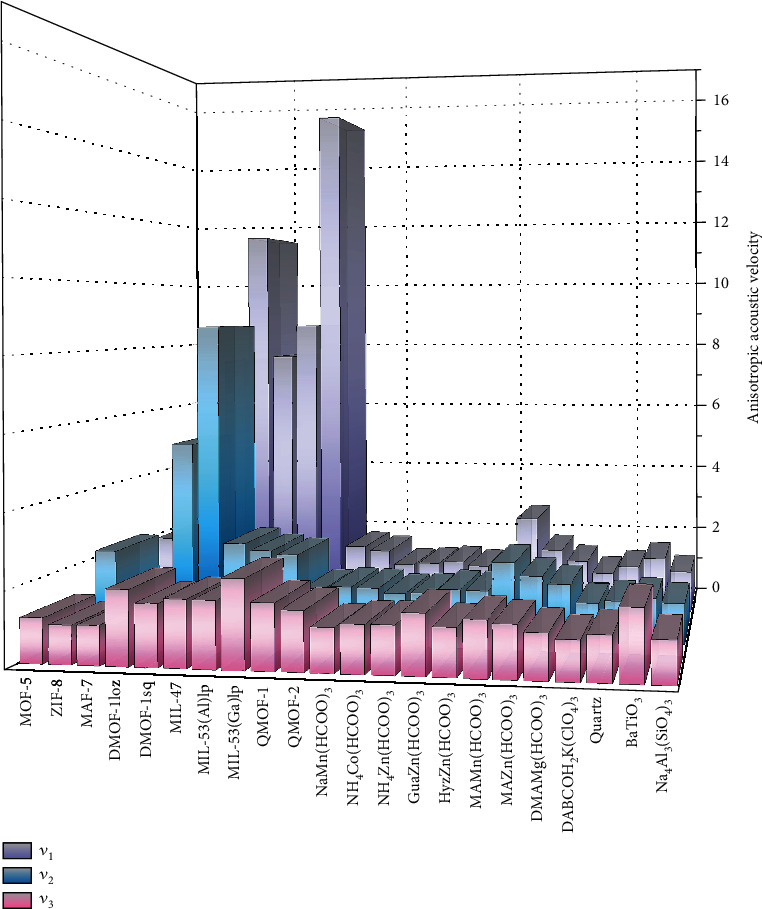
Ratios of the maximum and minimum velocities of three types of acoustic waves travelling in the 22 materials.

The ratios of the maximum and minimum velocities of each type of wave for these 22 materials were calculated as indicators to evaluate their anisotropy (A_*v*_ = *v*_max_/*v*_min_), and the results are shown in Figure 3. Notably, the *A*_*v*_ values of DMOF-1loz, DMOF-1sq, MIL-47, MIL-53(Al)lp, and MIL-53(Ga)lp are up to an order of magnitude higher than those of the 3 inorganic materials, which could give rise to some potential applications unavailable in conventional materials, as discussed below. In particular, MIL-53(Ga)lp has the highest acoustic velocity anisotropy, with anisotropy indices of the longitudinal wave velocity (*A*_*v*3_) and pure shear wave velocity (*A*_*v*1_) of 2.35 and 15.17, respectively.

The 3D surface contours and projected 2D plots of the acoustic velocities for MOF-5 are displayed in Fig. S1, which demonstrates that its *v*_1_, *v*_2_, and *v*_3_ are in the ranges of 1.67-3.13, 1.67-3.67, and 5.50-6.83 km/s, respectively. The *v*_1min_ and *v*_2min_ of MOF-5 are approximately half of those of BaTiO_3_, which also has a ReO_3_-related structure, while the *v*_1max_ and *v*_2max_ are close to those of BaTiO_3_. In addition, both the *v*_3max_ and *v*_3min_ values of MOF-5 approximate those of BaTiO_3_. These findings disclose that acoustic waves can propagate similarly in porous MOFs and dense oxides. Furthermore, the anisotropy of the 3 types of wave velocities in MOF-5 is also distinct, and *A*_*v*3_ (1.19) is significantly smaller than *A*_*v*1_ (1.88) and *A*_*v*2_ (2.19). As the Young's modulus of MOF-5 is determined by the connectivity of organic ligands, the isotropic periodic extension of the organic linkers in the structure leads to an isotropic Young's modulus corresponding to a small *A*_*v*3_. However, unlike the p-wave velocity, the s-wave velocity in MOF-5 is affected by the shear modulus. For the square-shaped building block in the structure, it is much more easily sheared along the organic linker direction (edge direction) than along the diagonal direction, hence resulting in dramatic anisotropy in *v*_1_ and *v*_2_ [32].

The 3D surface contours and 2D plots of the acoustic velocities for ZIF-8 and MAF-7 are displayed in Figs. S2 and S3. Although they are isomorphic with the sodalite structure, the *v*_1_, *v*_2_, and *v*_3_ of ZIF-8 are all slightly smaller than those of MAF-7. According to formulas (1)–(3), the difference is attributed to the enhanced Young's modulus of MAF-7 due to the electron-donating effect and its lower density (ZIF-8, 1.14 g/cm^3^; MAF-7, 1.08 g/cm^3^). In addition, the *v*_1_ and *v*_2_ values of ZIF-8 and MAF-7 are only approximately 26-31% of those of sodalite, while the *v*_3_ values are approximately 53-62% of the value of sodalite. These phenomena arise from the more obvious difference in the Young's moduli between ZIF-8/MAF-7 and sodalite than that in the shear moduli. Moreover, both ZIF-8 and MAF-7 exhibit very small anisotropy in sound velocities, which is reminiscent of that of sodalite.

DMOFs have unusual guest-dependent dynamic behaviour: the framework shrinks to a lozenge (DMOF-1loz) when the guest is present and expands to a square (DMOF-1sq) when the guest is released. Along with the change in unit cell volume from 1147 (DMOF-1sq) to 1114 Å^3^ (DMOF-1loz), the density increases from 0.826 to 0.850 g/cm^3^. This drastic structural change also leads to a significant difference in framework stiffness and corresponding acoustic velocities (Figs. S4 and S5). The *v*_1max_, *v*_2max_, *v*_3max_, and *v*_3min_ values of DMOF-1lsq are all larger than those of DMOF-1loz, while the *v*_1min_ and *v*_2min_ exhibit an inverse trend. Notably, the anisotropies in *v*_1_ and *v*_2_ of both the lozenge and square phases are very high, which are 7.55 and 11.43 for the former and 5.31 and 8.69 for the latter.

**Figure 4:**
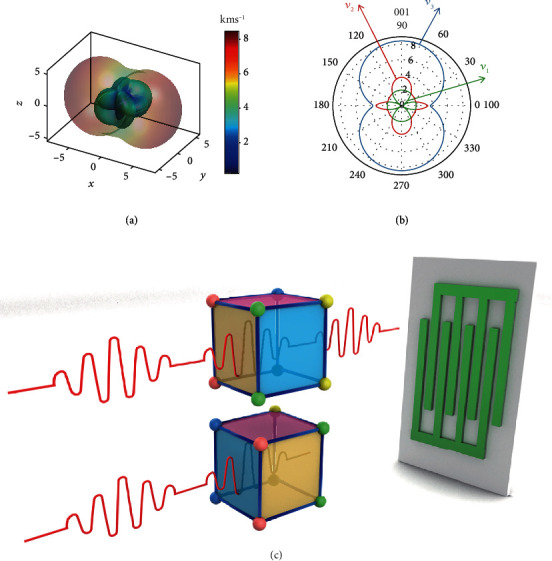
3D surfaces (a) and 2D polar plots (b) of the acoustic velocity for MIL-53(Ga)lp projected normal to the (010) plane. (c) Acoustic switching enabled by the high anisotropy in acoustic waves of MIL-53(Ga)lp: when the same sound waves pass through the material, the output sound wave velocities will attenuate by very different magnitudes.

The members of the MIL family of MOFs have similar structures but different densities (MIL-47, 1.01 g/cm^3^ < MIL − 53(Al)lp, 1.32 g/cm^3^ < MIL − 53(Ga)lp, 1.57 g/cm^3^), which leads to acoustic velocities in the same sequence as the density (Figures 4(a) and 4(b), S6, S7). For the Al and Ga analogues of MIL-53, the strength difference in the shorter Al-O and longer Ga-O coordination bonds is responsible for their different moduli and corresponding acoustic velocities. In addition, the anisotropy in *v*_1_ for all three frameworks is significantly high and is 7.69, 8.65, and 15.17 for MIL-47, MIL-53(Al)lp, and MIL-53(Ga)lp, respectively. In particular, the anisotropy in *v*_1_ of MIL-53Ga is the highest among all 22 materials, and such a large anisotropy is even larger than those of many 2D crystals. In terms of potential applications, MIL-53(Ga)lp could be utilized as a new type of acoustic switch through which acoustic waves can travel approximately 14 times faster in one direction than in the other, as shown in Figure 4(c).

QMOF-1 and QMOF-2 are constructed by ZnO and InO chains with isonicotinate and terephthalate linkers, respectively. The bond length of Zn-O is shorter than that of In-O, so the elastic moduli of QMOF-1 are larger than those of QMOF-2. In addition, the density of QMOF-1 (1.52 g/cm^3^) is less than that of QMOF-2 (1.76 g/cm^3^). Both aspects result in 53-65%, 101-111%, and 39-55% higher *v*_1_, *v*_2_, and *v*_3_ of QMOF-1 than those of QMOF-2 (Figs. S8, S9). Furthermore, the anisotropies in acoustic velocities of both MOFs are small, with the highest value less than 1.8. The *v*_1_, *v*_2_, and *v*_3_ values of QMOF-2 are approximately 47-58%, 48-57%, and 57-77% of those of quartz. However, the *v*_2max_, *v*_2min_, and *v*_3max_ values are approximately 15%, 2%, and 8% higher than those of quartz, although the *v*_1max_, *v*_1min_, and *v*_3min_ values are approximately 4%, 28%, and 20% lower.

As shown in Figures 2 and S10-17, the *v*_1min_ values of all formate frameworks are generally larger than those of porous MOFs and are broadly similar to that of the most structurally related BaTiO_3_. Taking [DMA][Mg(HCOO)_3_] as an example, its *v*_1_, *v*_2_, and *v*_3_ are approximately 101-110%, 72-92%, and 93-146% of those of BaTiO_3_. In addition, these formates generally have small anisotropy indices below 1.8 apart from [MA][Mn(HCOO)_3_] (*A*_*v*1_ = 2.71, *A*_*v*2_ = 2.12). The organic-inorganic framework DABCOH_2_K(ClO_4_)_3_ has a density of 3.15 g/cm^3^, which is much higher than those of all aforementioned MOFs. Considering the similar elastic moduli of DABCOH_2_K(ClO_4_)_3_ and perovskite formates, its acoustic velocities are generally smaller (Fig. S18). Compared with BaTiO_3_, its *v*_1_, *v*_2_, and *v*_3max_ are approximately 57-83%, 48-60%, and 52% lower, although its *v*_3min_ is almost the same.

### 2.3. Acoustic Impedance

Acoustic impedance (*Z*) describes the ratio of acoustic pressure to sound flow propagating in a material, which can be defined as the product of the acoustic velocity and the density of the material: *Z* = *v*∗*ρ*, where *v* and *ρ* are the acoustic velocity and density, respectively. The acoustic impedance can also be divided into transverse acoustic impedance and longitudinal acoustic impedance according to the different acoustic velocities. The longitudinal acoustic impedance is widely used in practice, so we calculated the maximum and minimum longitudinal acoustic impedances of these 22 materials.

**Figure 5:**
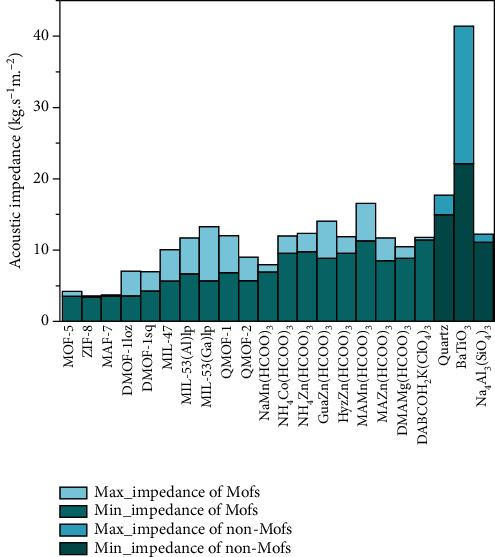
Maximum and minimum acoustic impedances of the 22 materials.

As seen from Figure 5, the longitudinal acoustic impedances of MOF-5, ZIF-8, and MAF-7 have very similar values and are the smallest among all 22 materials. The values of ZIF-8 (*Z*_min_ = 3.46, *Z*_max_ = 3.55 MPa∙s∙m^−1^) and MAF-7 (*Z*_min_ = 3.54, *Z*_max_ = 3.67 MPa∙s∙m^−1^) are only approximately 30% of those of their inorganic counterpart, sodalite (*Z*_min_ = 11.1, *Z*_max_ = 12.0 MPa∙s∙m^−1^), which is attributed to their much lower elastic moduli and density. For the MIL family, their density and *v*_3max_ increase in the following sequence: MIL − 47 < MIL − 53(Al)l < MIL − 53(Ga)lp, which leads to the same trend in their acoustic impedances: MIL-47 (*Z*_max_ = 10.04 MPa∙s∙m^−1^) < MIL-53(Al)lp (*Z*_max_ = 11.70 MPa∙s∙m^−1^) < MIL-53(Ga)lp (*Z*_max_ = 13.28 MPa∙s∙m^−1^). Both QMOF-1 and QMOF-2 have quartz-like structures, so the study of their acoustic impedances is of great significance for their application in piezoelectric sensors. The *Z*_min_ and *Z*_max_ of QMOF-1 and QMOF-2 are approximately 46% and 68% and approximately 38% and 51% of those of quartz, respectively. For the metal formates, their *Z*_min_ and *Z*_max_ values are in the range of 3.72-5.26 and 4.29-9.17 MPa∙s∙m^−1^. Taking the DMAMg(HCOO)_3_ perovskite as an example, its *Z*_min_ and *Z*_max_ are approximately 45% and 40% of those of the perovskite oxide BaTiO_3_. By comparison, it can be seen that the acoustic impedances of MOFs are generally lower than those of traditional inorganic materials.

### 2.4. Piezoelectric Properties

The acoustic and piezoelectric properties of materials are closely related since acoustic waves can be detected by piezoelectric materials or vice versa. According to the type of energy conversion, the piezoelectric effects can be divided into direct and indirect effects. The direct piezoelectric effect occurs when a piezoelectric material generates a dipole moment under external stress, while the indirect piezoelectric effect refers to the strain generated in piezoelectric materials when they are placed in an electric field [35].

**Figure 6:**
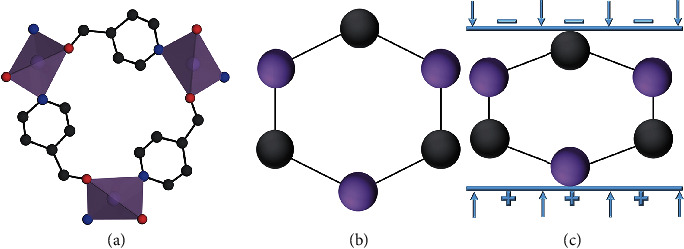
Schematic diagram illustrating the atomic origin of the piezoelectricity of QMOF-1: (a) crystal structure of QMOF-1; (b, c) equivalent diagrams of QMOF-1 without and with stress, respectively.

Among the 19 crystals, QMOF-1, QMOF-2, and DMAMg(HCOO)_3_ have piezoelectric properties owing to their 3, 622, and *m* noncentrosymmetric point groups, respectively. The voltage signal harvested by our experiment also confirmed the piezoelectric property of QMOF-1 (Fig. S22). Because of the structural similarity, the origin of the piezoelectric effect of QMOF-1 is reminiscent of that of quartz. As shown in Figure 6, Zn-O polyhedra and isonicotinate linkers are considered negatively and positively charged components, respectively. In the absence of stress, the positive and negative charge centres overlap, which leads to no macroscopic spontaneous polarization. However, negative and positive charges are generated on the top and bottom surfaces of the crystal, respectively, when the *a*-axis is stressed.

To further analyse the piezoelectric properties of QMOF-1, QMOF-2, and DMAMg(HCOO)_3_, the density functional perturbation theory (DFPT) calculation method is used to obtain their piezoelectric tensors. It is well known that the piezoelectric stress tensor [*e*], elastic compliance matrix [*s*], and piezoelectric strain tensor [*d*] can be related *via* the following formula: [*d*] = [*e*][*s*] [36, 37]. The full piezoelectric stress and strain tensors of all three MOFs are listed in Tables S2, S4, and S5. In all 3 MOFs, the ionic contribution is much larger than the electronic contribution to the piezoelectric stress tensors. For QMOF-1, the piezoelectric strain tensor *d*_14_ (23.64 pC/N) is 31.3 times larger than that of quartz (*d*_14_ = 0.73 pC/N), and its *d*_11_ is 4.60 pC/N. QMOF-2 only has a *d*_14_ tensor (2.52 pC/N) due to its 622 symmetry, which is approximately 3.5 times higher than that of quartz. The *d*_33_ and *d*_15_ values of DMAMg(HCOO)_3_ are 3.19 and -10.5 pC/N, respectively, which are only approximately 2% and 4% of those of the commercial piezoelectric perovskite oxide BaTiO_3_. In addition, the *d*_33_ value of DMAMg(HCOO)_3_ is 2-3 orders of magnitude lower than those of high-performance hybrid perovskite piezoelectrics, such as [TMCM][MnCl_3_] (TMCM = trimethylchloromethyl ammonium, *d*_33_ = 186 pC/N) and (TMFM)_x_(TMCM)_1–x_CdCl_3_ (TMFM = trimethylfluoromethyl ammonium, *d*_33_ = 1540 pC/N) [38, 39].

The piezoelectric strain tensor *d* in these 3 MOFs is mainly affected by two factors: the magnitude of the atomic displacement caused by the strain and the charge change caused by the atomic displacement. The Born effective charge (BEC) is defined as the change in electron polarization caused by ion displacement and is the second derivative of the energy with respect to the displacement and electric field. In this regard, the BEC (*Z*_*mα*_) can directly reflect the contribution of the second factor to the piezoelectric strain tensor. The BEC can be expressed as:

(1) $$\begin{array}{c} Z_{m\alpha }=-\Omega_{0}\frac{\partial^{2}E}{\partial \mu_{m}\partial \sigma_{\alpha }}\left|\eta \right., \end{array}$$

where *μ*, *σ*, and *η* represent the atomic displacement, electric field, and strain, respectively. *E* denotes the total energy of the material in the ground state, and _0_ is the unit cell volume of the material.

To explore the direction-dependent piezoelectric effects, the BEC of the zinc ions along different directions in QMOF-1 was calculated, and the results are listed in Table S3. There are three nonequivalent zinc ions in the unit cell of QMOF-1, and the sum of their BECs along the *c*-axis is approximately half of those along the *a*/*b*-axis. As the zinc ion normally shows a positive divalence, it is prone to exhibit charge transfer if its BEC is larger than 2. The BECs of the three zinc elements are 1.59, 1.59, and 1.58 along the *c*-axis, while they are 2.11, 3.79, and 2.33 along the *a*-axis and 3.38, 1.69, and 3.16 along the *b*-axis. Such a significant difference indicates that transfer of their charges is more difficult along the *c*-axis than along the *a*/*b*-axis, hence giving rise to smaller *d*_33_ but larger *d*_11_ and *d*_22_.

## 3. Discussion

Our above analyses demonstrate the tight structural-acoustic property relationships of MOFs and suggest some tuning approaches *via* crystal engineering. First, variation of the organic ligands can result in prominent differences in the elastic moduli of MOFs [14] and the corresponding acoustic velocities and impedance for the same structural topology and similar densities. As this strategy has been well demonstrated by the difference in acoustic properties between the prototypical ZIF-8 and MAF-7 by simply varying the ligand, it could be widely applied in other MOF systems. Second, metal nodes play an important role in determining the framework rigidity and therefore substantially influence the corresponding acoustic properties. For the MIL-53 family, the Al analogue is stiffer than the Ga counterpart due to the shorter coordination bonds [32], which leads to its higher sound velocities but lower acoustic anisotropy. Similarly, the Jahn-Teller effect and ligand splitting stabilization energy could be employed to modulate the elasticity and corresponding acoustic properties of MOFs [40]. Furthermore, the coordination number of metal nodes would be an alternative way to tune the acoustic properties of MOFs. For instance, MOFs with highly coordinated metal nodes show higher resistance towards shear stress than those with low-coordinated structures and therefore exhibit higher transverse wave velocities. Third, the acoustic properties of MOFs could also be tuned and engineered by varying host-guest interactions. For instance, the much stronger hydrogen bonding in [GUA][Mn(HCOO)_3_] leads to twice the Young's moduli compared to those of the analogous [AZE][Mn(HCOO)_3_] (AZE = azetidinium), which naturally results in its much larger acoustic velocities and impedance [41]. On the other hand, gas molecule-filled MOFs exhibit lower acoustic velocities owing to unaltered elastic moduli but significantly increased density compared to their parent phases [42].

In terms of the piezoelectricity of MOFs, very few studies have been reported, and more research efforts need to be devoted to this field, especially considering the availability of vast numbers of noncentrosymmetric MOFs with diverse structures and topologies. When sufficient piezoelectric MOFs are identified and their structure-property relationship is corroborated, the next goal would be to optimize their performance to catch up with those of their conventional ceramic counterparts. In this regard, screening more noncentrosymmetric MOFs using DFPT calculations with the aid of artificial intelligence would be a promising method if combined with complementary experimental confirmation.

## 4. Conclusions

In conclusion, the acoustic properties of 19 MOFs were systematically investigated *via* density functional theory (DFT) calculations and by solving the Christoffel equations. Our results show that the MOFs have a very wide range of acoustic velocities due to their structural diversity and abundant chemical compositions. Meanwhile, some MOFs can exhibit very high anisotropy in acoustic velocities, which could be applicable in acoustic switches. In addition, the low densities and moduli of MOFs are responsible for their small acoustic impedances compared with their inorganic counterparts. Furthermore, the piezoelectric properties, which are closely related to the acoustic properties, were calculated for two quartz-like and one perovskite-like MOFs. Our results reveal that these MOFs can exhibit significant piezoelectricity, which mainly originates from the ionic contribution. Finally, we propose some strategies for engineering the acoustic properties of MOFs and discuss future directions for studying the piezoelectricity of MOFs. This work offers an original study of the fundamental acoustic properties of MOFs, which we believe could inspire further attention in this largely unexplored area.

## 5. Methods

Structural optimizations and calculations of elastic constants based on DFT were performed using a plane-wave basis set as implemented in the Vienna ab initio simulation package (VASP) [43–45]. Projector augmented wave (PAW) pseudopotentials were employed to describe ion-electron interactions [46]. The considered PAW pseudopotentials and numbers of valence electrons were H (1), C (4), N (5), O (6), Na_pv (7), Mg (2), Al (3), Si (4), Cl (7), K_sv (9), Ca_sv (10), Ti_sv (12), V_sv (13), Mn_pv (13), Fe (8), Co (9), Cu (11), Zn (12), Ga_d (13), and Ba_sv (10). The GGA (PBEsol) functional was used to further accurately describe the interactions within each MOF structure. Considering the abundant van der Waals interactions existed in MOF systems, dispersion corrected calculations are known to be crucial in the correct optimization of their crystal structures. Specifically, GGA (PBEsol) underestimates the binding energy, hence resulting in larger lattice parameters for these molecular systems. By applying the dispersion corrections, more reasonable binding energies could be obtained, which gives rise to more reliable lattice parameters [47, 48]. As elastic constants are obtained by the second derivative of the total energy with respective to specific strain and strongly correlated to the relative change of the crystal structure, obtaining accurate cell parameters are of vital importance in the calculations. In this regard, the van der Waals corrected zero damping DFT-D3 method was chosen in our calculations. Considering that the calculation of elastic constants requires a high standard for the structures of materials, the atomic positions and cell parameters were completely relaxed when the structure was optimized. The elastic constants were calculated by setting six finite distortions of the lattice and the step size to 0.015 Å. The total energy converged to within 10^−7^ eV, and the residual forces on each atom were less than 0.005 eV/Å. The energy cutoff for the plane-wave basis and the smallest allowed spacing between *k*-points of these compounds were set to 500 eV and 0.5 Å^−1^, respectively.

The acoustic velocity was obtained by solving the Christoffel equation with the material density and the elastic constants calculated by DFT [49]. The Christoffel equation can be expressed as:

(2) $$\begin{array}{c} \Gamma u=\rho v^{2}u, \end{array}$$

(3) $$\begin{array}{c} \left(\begin{array}{ccc} \Gamma_{11}-\rho v^{2} & \Gamma_{12} & \Gamma_{13}\\ \Gamma_{21} & \Gamma_{22}-\rho v^{2} & \Gamma_{23}\\ \Gamma_{31} & \Gamma_{32} & \Gamma_{33}-\rho v^{2} \end{array}\right)=0, \end{array}$$

(4) $$\begin{array}{c} \Gamma_{ij}=Cijkl*\alpha_{k}\alpha_{l}, \end{array}$$

where *u* is the elastic displacement field and Γ, *v*, *ρ*, and *α* are the Christoffel matrix, acoustic velocity in the crystal, density, and direction vector, respectively.

The second derivative of the total energy reflects many properties of the calculated structure, such as the Born effective charge (BEC) and piezoelectric and dielectric tensors. The density functional perturbation theory proposed by Baroni et al. in 1987 can effectively calculate the derivative of the total energy with respect to strain, atomic displacement, and electric field [50–53]. The expression for the total energy *E* (*μ*, *σ*, *η*) under perturbation is as follows:

(5) $$\begin{array}{c} E\left(\mu ,\sigma ,\eta \right)\frac{1}{\Omega_{0}}\left[E_{0}-\Omega *\sigma *P\right], \end{array}$$

where *μ*, *σ*, and *η* represent the atomic displacement, electric field, and strain, respectively. *E* (*μ*, *σ*, *η*) represents the total energy of the material in the ground state, _0_ is the unit cell volume of the material and is the unit cell volume of the material under perturbation, and *P* is the electric field polarization.

The piezoelectric effect reflects the deformation degree of a material under an electric field, which can be evaluated by the piezoelectric stress tensor (*e*_*αj*_). The piezoelectric tensors calculated by DFPT are divided into the electronic contribution (${\bar{e}}_{\alpha j}$) and ionic contribution (${\hat{e}}_{\alpha j}$). The former is an ion-clamped piezoelectric tensor that ignores the effects of atomic relaxation; in contrast, the latter takes into account the effect of the displacement of atoms under strain on the piezoelectric tensor. Therefore, the piezoelectric tensor can be expressed as:

(6) $$\begin{array}{c} e_{\alpha j}=\frac{\partial P_{\alpha }}{\partial \eta_{j}}={\bar{e}}_{\alpha j}+{\hat{e}}_{\alpha j}, \end{array}$$

(7) $$\begin{array}{c} {\bar{e}}_{\alpha j}=\frac{\partial^{2}E}{\partial \sigma_{\alpha }\partial \eta_{J}}\left|\mu \right., \end{array}$$

(8) $$\begin{array}{c} {\hat{e}}_{\alpha j}=Z_{\alpha \beta }\frac{\partial \mu_{\beta }}{\partial \eta_{j}}. \end{array}$$

In this part of the DFPT calculation, we used the previously highly optimized crystal structure as the calculation model and improved the self-consistent field convergence standard to 10^−8^ eV.

## Data Availability

All data needed in the paper are present in the paper and in the supplementary section. Additional data related to this paper may be requested from the authors.

## Supplementary Materials

Supplementary Materials Table S1: summary of the density *ρ* (g/cm^3^) and elastic constants *C*_*ij*_ (GPa) of the MOFs and inorganic materials obtained from the DFT calculations. Table S2: summary of the transverse wave velocities and longitudinal wave velocities (m/s) of the MOFs and inorganic materials. Table S3: clamped-ion $\bar{\text{e}}$ (C/m^2^), relaxed-ion $\hat{e}$ (C/m^2^), and piezoelectric strain tensor *d* (pC/N) for QMOF-1. Table S4: summary of the BEC of QMOF-1 obtained from the DFPT calculation. Table S5: clamped-ion $\bar{e}$ (C/m^2^), relaxed-ion $\hat{e}$ (C/m^2^), and piezoelectric strain tensor *d* (pC/N) for QMOF-2. Table S6: clamped-ion $\bar{e}$ (C/m^2^), relaxed-ion ê (C/m^2^), and piezoelectric strain tensor *d* (pC/N) for DMAMg(HCOO)_3_. Figure S1: (a) 3D surfaces of the acoustic velocity for MOF-5. (b–d) 2D plots of the acoustic velocity for MOF-5 projected normal to the (100), (010), and (001) planes, respectively. The green, red, and blue lines represent *v*_1_, *v*_2_, and *v*_3_, respectively. Figure S2: (a) 3D surfaces of the acoustic velocity for ZIF-8. (b–d) 2D plots of the acoustic velocity for ZIF-8 projected normal to the (100), (010), and (001) planes, respectively. The green, red, and blue lines represent *v*_1_, *v*_2_, and *v*_3_, respectively. Figure S3: (a) 3D surfaces of the acoustic velocity for MAF-7. (b–d) 2D plots of the acoustic velocity for MAF-7 projected normal to the (100), (010), and (001) planes, respectively. The green, red, and blue lines represent *v*_1_, *v*_2_, and *v*_3_, respectively. Figure S4: (a) 3D surfaces of the acoustic velocity for DMOF-1loz. (b–d) 2D plots of the acoustic velocity for DMOF-1loz projected normal to the (100), (010), and (001) planes, respectively. The green, red, and blue lines represent *v*_1_, *v*_2_, and *v*_3_, respectively. Figure S5: (a) 3D surfaces of the acoustic velocity for DMOF-1sq. (b–d) 2D plots of the acoustic velocity for DMOF-1sq projected normal to the (100), (010), and (001) planes, respectively. The green, red, and blue lines represent *v*_1_, *v*_2_, and *v*_3_, respectively. Figure S6: (a) 3D surfaces of the acoustic velocity for MIL-47. (b–d) 2D plots of the acoustic velocity for MIL-47 projected normal to the (100), (010), and (001) planes, respectively. The green, red, and blue lines represent *v*_1_, *v*_2_, and *v*_3_, respectively. Figure S7: (a) 3D surfaces of the acoustic velocity for MIL-53(Al)lp. (b–d) 2D plots of the acoustic velocity for MIL-53(Al)lp projected normal to the (100), (010), and (001) planes, respectively. The green, red, and blue lines represent *v*_1_, *v*_2_, and *v*_3_, respectively. Figure S8: (a) 3D surfaces of the acoustic velocity for QMOF-1. (b–d) 2D plots of the acoustic velocity for QMOF-1 projected normal to the (100), (010), and (001) planes, respectively. The green, red, and blue lines represent *v*_1_, v_2_, and *v*_3_, respectively. Figure S9: (a) 3D surfaces of the acoustic velocity for QMOF-2. (b–d) 2D plots of the acoustic velocity for QMOF-2 projected normal to the (100), (010), and (001) planes, respectively. The green, red, and blue lines represent *v*_1_, *v*_2_, and *v*_3_, respectively. Figure S10: (a) 3D surfaces of the acoustic velocity for NaMn(HCOO)_3_. (b–d) 2D plots of the acoustic velocity for NaMn(HCOO)_3_ projected normal to the (100), (010), and (001) planes, respectively. The green, red, and blue lines represent *v*_1_, *v*_2_, and *v*_3_, respectively. Figure S11: (a) 3D surfaces of the acoustic velocity for NH_4_Co(HCOO)_3_. (b–d) 2D plots of the acoustic velocity for NH_4_Co(HCOO)_3_ projected normal to the (100), (010), and (001) planes, respectively. The green, red, and blue lines represent *v*_1_, *v*_2_, and *v*_3_, respectively. Figure S12: (a) 3D surfaces of the acoustic velocity for NH_4_Zn(HCOO)_3_. (b–d) 2D plots of the acoustic velocity for NH_4_Zn(HCOO)_3_ projected normal to the (100), (010), and (001) planes, respectively. The green, red, and blue lines represent *v*_1_, *v*_2_, and *v*_3_, respectively. Figure S13: (a) 3D surfaces of the acoustic velocity for GuaZn(HCOO)_3_. (b–d) 2D plots of the acoustic velocity for GuaZn(HCOO)_3_ projected normal to the (100), (010), and (001) planes, respectively. The green, red, and blue lines represent *v*_1_, *v*_2_, and *v*_3_, respectively. Figure S14: (a) 3D surfaces of the acoustic velocity for HyzZn(HCOO)_3_. (b–d) 2D plots of the acoustic velocity for HyzZn(HCOO)_3_ projected normal to the (100), (010), and (001) planes, respectively. The green, red, and blue lines represent *v*_1_, *v*_2_, and *v*_3_, respectively. Figure S15: (a) 3D surfaces of the acoustic velocity for MAMn(HCOO)_3_. (b–d) 2D plots of the acoustic velocity for MAMn(HCOO)_3_projected normal to the (100), (010), and (001) planes, respectively. The green, red, and blue lines represent *v*_1_, *v*_2_, and *v*_3_, respectively. Figure S16: (a) 3D surfaces of the acoustic velocity for MAZn(HCOO)_3_. (b–d) 2D plots of the acoustic velocity for MAZn(HCOO)_3_ projected normal to the (100), (010), and (001) planes, respectively. The green, red, and blue lines represent *v*_1_, *v*_2_, and *v*_3_, respectively. Figure S17: (a) 3D surfaces of the acoustic velocity for DMAMg(HCOO)_3_. (b–d) 2D plots of the acoustic velocity for DMAMg(HCOO)_3_ projected normal to the (100), (010), and (001) planes, respectively. The green, red, and blue lines represent *v*_1_, *v*_2_, and *v*_3_, respectively. Figure S18: (a) 3D surfaces of the acoustic velocity for DABCOH_2_K(ClO_4_)_3_. (b–d) 2D plots of the acoustic velocity for DABCOH_2_K(ClO_4_)_3_ projected normal to the (100), (010), and (001) planes, respectively. The green, red, and blue lines represent *v*_1_, *v*_2_, and *v*_3_, respectively. Figure S19: (a) 3D surfaces of the acoustic velocity for quartz. (b–d) 2D plots of the acoustic velocity for quartz projected normal to the (100), (010), and (001) planes, respectively. The green, red, and blue lines represent *v*_1_, *v*_2_, and *v*_3_, respectively. Figure S20: (a) 3D surfaces of the acoustic velocity for BaTiO_3_. (b–d) 2D plots of the acoustic velocity for BaTiO_3_ projected normal to the (100), (010), and (001) planes, respectively. The green, red, and blue lines represent *v*_1_, *v*_2_, and *v*_3_, respectively. Figure S21: (a) 3D surfaces of the acoustic velocity for Na_4_Al_3_(SiO_4_)_3_. (b–d) 2D plots of the acoustic velocity for Na_4_Al_3_(SiO_4_)_3_ projected normal to the (100), (010), and (001) planes, respectively. The green, red, and blue lines represent *v*_1_, *v*_2_, and *v*_3_, respectively. Figure S22: (a) Schematic diagram of piezoelectric energy harvester based on polycrystalline pressed samples of QMOF-1. (b) Generated output voltages for QMOF-1 under certain mechanical press recorded by digital oscilloscope. The experiments were conducted based on a sandwich-structured Cu electrode-polycrystalline samples-Cu electrode device, under a periodic impact created by a mechanical contactor. The output voltage was measured using an oscilloscope (Keysight DSOX3024T).

## References

[1] Cheetham A. K., Rao C. N. R., Feller R. K. (2006). Structural diversity and chemical trends in hybrid inorganic–organic framework materials. *Chemical Communications*.

[2] Rao C. N. R., Cheetham A. K., Thirumurugan A. (2008). Hybrid inorganic–organic materials: a new family in condensed matter physics. *Journal of Physics-Condensed Matter*.

[3] Cheetham A. K., Rao C. N. R. (2007). There’s room in the middle. *Science*.

[4] Hu Z. C., Deibert B. J., Li J. (2014). Luminescent metal-organic frameworks for chemical sensing and explosive detection. *Chemical Society Reviews*.

[5] Coronado E. (2020). Molecular magnetism: from chemical design to spin control in molecules, materials and devices. *Nature Reviews Materials*.

[6] Jain P., Stroppa A., Nabok D. (2016). Switchable electric polarization and ferroelectric domains in a metal- organic-framework. *Npj Quantum Materials*.

[7] Stroppa A., Barone P., Jain P., Perez-Mato J. M., Picozzi S. (2013). Hybrid improper ferroelectricity in a multiferroic and magnetoelectric metal-organic framework. *Advanced Materials*.

[8] Li B., Wen H. M., Cui Y. J., Zhou W., Qian G., Chen B. (2016). Emerging multifunctional metal-organic framework materials. *Advanced Materials*.

[9] Miller Q. R. S., Nune S. K., Schaef H. T. (2018). Microporous and flexible framework acoustic metamaterials for sound attenuation and contrast agent applications. *ACS Applied Materials Interfaces*.

[10] Ahmed H., Rezk A. R., Richardson J. J. (2019). Acoustomicrofluidic assembly of oriented and simultaneously activated metal- organic frameworks. *Nature Communications*.

[11] Leong K., Foster M. E., Wong B. M. (2014). Energy and charge transfer by donor-acceptor pairs confined in a metal-organic framework: a spectroscopic and computational investigation. *Journal of Materials Chemistry A*.

[12] Haussühl S. (2007). *Physical properties of crystals: an introduction*.

[13] Tan J. C., Cheetham A. K. (2011). Mechanical properties of hybrid inorganic–organic framework materials: establishing fundamental structure–property relationships. *Chemical Society Reviews*.

[14] Li W., Henke S., Cheetham A. K. (2014). Research update: mechanical properties of metal-organic frameworks – influence of structure and chemical bonding. *APL Materials*.

[15] Heinen J., Burtch N. C., Walton K. S., Dubbeldam D. (2017). Flexible force field parameterization through fitting on the ab initio-derived elastic tensor. *Journal of Chemical Theory and Computation*.

[16] Xu W., Tu B., Liu Q. (2020). Anisotropic reticular chemistry. *Nature Reviews Materials*.

[17] Rowsell J. L. C., Millward A. R., Park K. S., Yaghi O. M. (2004). Hydrogen sorption in functionalized metal−organic frameworks. *Journal of the American Chemical Society*.

[18] Park K. S., Ni Z., Cote A. P. (2006). Exceptional chemical and thermal stability of zeolitic imidazolate frameworks. *Proceedings of the National Academy of Sciences of the United States of America*.

[19] Zhang J. P., Zhu A. X., Lin R. B., Qi X. L., Chen X. M. (2011). Pore surface tailored SOD-type metal-organic zeolites. *Advanced Materials*.

[20] Dybtsev D. N., Chun H., Kim K. (2004). Rigid and flexible: a highly porous metal–organic framework with unusual guest-dependent dynamic behavior. *Angewandte Chemie-International Edition*.

[21] Yot P. G., Ma Q., Haines J. (2012). Large breathing of the MOF MIL-47(VIV) under mechanical pressure: a joint experimental–modelling exploration. *Chemical Science*.

[22] Liu Y., Her J. H., Dailly A., Ramirez-Cuesta A. J., Neumann D. A., Brown C. M. (2008). Reversible structural transition in MIL-53 with large temperature hysteresis. *Journal of the American Chemical Society*.

[23] Sun J., Weng L., Zhou Y. (2002). QMOF-1 and QMOF-2: three-dimensional metal–organic open frameworks with a quartzlike topology. *Angewandte Chemie-International Edition*.

[24] Eikeland E., Lock N., Filsø M. (2014). Alkali metal ion templated transition metal formate framework materials: synthesis, crystal structures, ion migration, and magnetism. *Inorganic Chemistry*.

[25] Xu G. C., Ma X. M., Zhang L., Wang Z. M., Gao S. (2010). Disorder−order ferroelectric transition in the metal formate framework of [NH_4_][Zn(HCOO)_3_]. *Journal of the American Chemical Society*.

[26] Xu G. C., Zhang W., Ma X. M. (2011). Coexistence of magnetic and electric orderings in the metal–formate frameworks of [NH_4_][M(HCOO)_3_]. *Journal of the American Chemical Society*.

[27] Wang Z., Zhang B., Otsuka T., Inoue K., Kobayashi H., Kurmoo M. (2004). Anionic NaCl-type frameworks of [Mn^II^(HCOO)^3−^], templated by alkylammonium, exhibit weak ferromagnetism. *Dalton Transactions*.

[28] Hu K. L., Kurmoo M., Wang Z. M., Gao S. (2009). Metal-organic perovskites: synthesis, structures, and magnetic properties of [C(NH_2_)_3_][MII(HCOO)_3_] (M=Mn, Fe, Co, Ni, Cu, and Zn; C(NH2)3= Guanidinium). *Chemistry - A European Journal*.

[29] Wang Z., Zhang X., Batten S. R., Kurmoo M., Gao S. (2007). [CH_3_NH_2_(CH_2_)_2_NH_2_CH_3_][M_2_(HCOO)_6_] (M = Mn^II^and Co^II^): weak ferromagnetic metal formate frameworks of unique binodal 6-connected (4^12^·6^3^)(4^9^·6^6^) topology, templated by a diammonium cation. *Inorganic Chemistry*.

[30] Chen S., Shang R., Hu K. L., Wang Z. M., Gao S. (2014). [NH_2_NH_3_][M(HCOO)_3_] (M = Mn^2+^, Zn^2+^, Co^2+^ and Mg^2+^): structural phase transitions, prominent dielectric anomalies and negative thermal expansion, and magnetic ordering. *Inorganic Chemistry Frontiers*.

[31] Jin Z. M., Pan Y. J., Li X. F., Hu M. L., Shen L. (2003). Diazabicyclo[2.2.2]octane-1,4-diium occluded in cubic anionic coordinated framework: the role of trifurcated hydrogen bonds of N-H⋯O and C-H⋯O. *Journal of Molecular Structure*.

[32] Ortiz A. U., Boutin A., Fuchs A. H., Coudert F. X. (2012). Anisotropic elastic properties of flexible metal-organic frameworks: how soft are soft porous crystals?. *Physical Review Letters*.

[33] Tan J. C., Civalleri B., Lin C. C. (2012). Exceptionally low shear modulus in a prototypical imidazole-based metal-organic framework. *Physical Review Letters*.

[34] Bahr D. F., Reid J. A., Mook W. M. (2007). Mechanical properties of cubic zinc carboxylate IRMOF-1 metal-organic framework crystals. *Physical Review B*.

[35] Nelson W. G. (2010). *Piezoelectric materials: structure, properties and applications*.

[36] Wang Z. X., Zhang H., Wang F. (2020). Superior transverse piezoelectricity in a halide perovskite molecular ferroelectric thin film. *Journal of the American Chemical Society*.

[37] Wang H., Liu H. H., Zhang Z. Y. (2019). Large piezoelectric response in a family of metal-free perovskite ferroelectric compounds from first-principles calculations. *Npj Computational Materials*.

[38] You Y. M., Liao W. Q., Zhao D. W. (2017). An organic-inorganic perovskite ferroelectric with large piezoelectric response. *Science*.

[39] Liao W. Q., Zhao D. W., Tang Y. Y. (2019). A molecular perovskite solid solution with piezoelectricity stronger than lead zirconate titanate. *Science*.

[40] Ji L. J., Sun S. J., Qin Y., Li K., Li W. (2019). Mechanical properties of hybrid organic-inorganic perovskites. *Coordination Chemistry Reviews*.

[41] Li W., Thirumurugan A., Barton P. T. (2014). Mechanical tunability via hydrogen bonding in metal-organic frameworks with the perovskite architecture. *Journal of the American Chemical Society*.

[42] Ba A., Kovalenko A., Aristégui C., Mondain-Monval O., Brunet T. (2017). Soft porous silicone rubbers with ultra-low sound speeds in acoustic metamaterials. *Scientific Reports*.

[43] Kresse G., Furthmüller J. (1996). Efficient iterative schemes forab initiototal-energy calculations using a plane-wave basis set. *Physical Review B*.

[44] Kresse G., Furthmüller J. (1996). Efficiency of ab-initio total energy calculations for metals and semiconductors using a plane-wave basis set. *Computational Materials Science*.

[45] Kresse G., Hafner J. (1993). Ab initiomolecular dynamics for liquid metals. *Physical Review B*.

[46] Blochl P. E. (1994). Projector augmented-wave method. *Physical Review B*.

[47] Grimme S., Antony J., Ehrlich S., Krieg H. (2010). A consistent and accurateab initioparametrization of density functional dispersion correction (DFT-D) for the 94 elements H-Pu. *Journal of Chemical Physics*.

[48] Chan B., Ball G. E. (2013). A benchmark ab initio and DFT study of the structure and binding of methane in the *σ*–alkane complex _CpRe(CO)2(CH4)_. *Journal of Chemical Theory and Computation*.

[49] Auld B. A. (1973). *Acoustic Fields and Waves in Solid*.

[50] Hamann D. R., Wu X., Rabe K. M., Vanderbilt D. (2005). Erratum: Metric tensor formulation of strain in density-functional perturbation theory. *Physical Review B*.

[51] Sakhya A. P., Dutta A., Shannigrahi S., Sinha T. P. (2016). Electronic structure, optical dielectric constant and born effective charge of EuAlO_3_. *Journal of Physics and Chemistry of Solids*.

[52] Filippetti A., Spaldin N. A. (2003). Strong-correlation effects in Born effective charges. *Physical Review B*.

[53] Baroni S. (1987). Green’s-function approach to linear response in solids. *Physical Review Letters*.

