# Supplementary material for: Acoustic Properties of Metal-Organic Frameworks
Source: Research (Wash D C). 2021 Jun 1;2021:9850151. doi: 10.34133/2021/9850151 (PMC8254136; doi:10.34133/2021/9850151)
Supplement: Supplementary Materials — Table S1: summary of the density ρ (g/cm3) and elastic constants Cij (GPa) of the MOFs and inorganic materials obtained from the DFT calculations. Table S2: summary of the transverse wave velocities and longitudinal wave velocities (m/s) of the MOFs and inorganic materials. Table S3: clamped-ion e¯ (C/m2), relaxed-ion e^ (C/m2), and piezoelectric strain tensor d (pC/N) for QMOF-1. Table S4: summary of the BEC of QMOF-1 obtained from the DFPT calculation. Table S5: clamped-ion e¯ (C/m2), relaxed-ion e^ (C/m2), and piezoelectric strain tensor d (pC/N) for QMOF-2. Table S6: clamped-ion e¯ (C/m2), relaxed-ion ê (C/m2), and piezoelectric strain tensor d (pC/N) for DMAMg(HCOO)3. Figure S1: (a) 3D surfaces of the acoustic velocity for MOF-5. (b–d) 2D plots of the acoustic velocity for MOF-5 projected normal to the (100), (010), and (001) planes, respectively. The green, red, and blue lines represent v1, v2, and v3, respectively. Figure S2: (a) 3D surfaces of the acoustic velocity for ZIF-8. (b–d) 2D plots of the acoustic velocity for ZIF-8 projected normal to the (100), (010), and (001) planes, respectively. The green, red, and blue lines represent v1, v2, and v3, respectively. Figure S3: (a) 3D surfaces of the acoustic velocity for MAF-7. (b–d) 2D plots of the acoustic velocity for MAF-7 projected normal to the (100), (010), and (001) planes, respectively. The green, red, and blue lines represent v1, v2, and v3, respectively. Figure S4: (a) 3D surfaces of the acoustic velocity for DMOF-1loz. (b–d) 2D plots of the acoustic velocity for DMOF-1loz projected normal to the (100), (010), and (001) planes, respectively. The green, red, and blue lines represent v1, v2, and v3, respectively. Figure S5: (a) 3D surfaces of the acoustic velocity for DMOF-1sq. (b–d) 2D plots of the acoustic velocity for DMOF-1sq projected normal to the (100), (010), and (001) planes, respectively. The green, red, and blue lines represent v1, v2, and v3, respectively. Figure S6: (a) 3D surfaces of the [file 9850151.f1.docx]

Supporting Information

Acoustic properties of Metal organic frameworks

Zhi-Gang Li^1, †^, Kai Li^1,^ ^†^, Li-Yuan Dong^2^, Tian-Meng Guo^1^, Muhammad Azeem^1^, Wei Li^1*^ and Xian-He Bu^1^

^1^School of Materials Science and Engineering, Tianjin Key Lab of Metal and Molecule-Based Material Chemistry, Nankai University, Tianjin 300350, China

^2^ School of Physics and Wuhan National Laboratory for Optoelectronics, Huazhong University of Science and Technology, Wuhan 430074, China

*Correspondence should be addressed to Wei Li; wl276@nankai.edu.cn

Xian-He Bu; [buxh@nankai.edu.cn](mailto:buxh@nankai.edu.cn)

^†^These authors contributed equally to this work

**Table S1.** Summary of the density *ρ* (g/cm^3^) and elastic constants *C_ij_* (GPa) of the MOFs obtained from the DFT calculations.

| **No.** | **Materials** | **Density** | ***C*_11_** | ***C*_12_** | ***C*_44_** | ***C*_13_** | ***C*_33_** | ***C*_22_** | ***C*_23_** | ***C*_55_** | ***C_66_*** | ***C_1_*_4_** | ***Crystal system*** | **Ref** |
| --- | --- | --- | --- | --- | --- | --- | --- | --- | --- | --- | --- | --- | --- | --- |
| 1 | MOF-5 | 0.61 | 28.50 | 12.10 | 1.70 | - | - | - | - | - | - | - | cubic | 1 |
| 2 | ZIF-8 | 1.14 | 11.04 | 8.33 | 0.94 | - | - | - | - | - | - | - | cubic | 2 |
| 3 | MAF-7 | 1.08 | 11.59 | 9.64 | 1.64 | - | - | - | - | - | - | - | cubic | 3 |
| 4 | DMOF-1loz | 1.67 | 57.2 | 9.9 | 0.6 | 31.4 | 17.7 | 35.6 | 5.5 | 16.4 | 0.7 | - | Orthorhombic | 4 |
| 5 | DMOF-1sq | 1.67 | 35.3 | 7.3 | 0.1 | 7.6 | 58.5 | 58.2 | 11.7 | 0.4 | 0.3 | - | Orthorhombic | 4 |
| 6 | MIL-47 | 1.01 | 40.7 | 12.6 | 50.8 | 9.3 | 36.2 | 62.6 | 47.0 | 7.8 | 9.3 | - | Orthorhombic | 4 |
| 7 | MIL-53(Al)lp | 1.32 | 90.9 | 20.4 | 7.2 | 54.3 | 33.3 | 65.6 | 12.4 | 39.5 | 8.3 | - | Orthorhombic | 4 |
| 8 | MIL-53(Ga)lp | 1.57 | 112.3 | 22.9 | 5.5 | 45.4 | 18.5 | 56.7 | 10.9 | 21.7 | 6.6 | - | Orthorhombic | 4 |
| 9 | QMOF-1 | 1.52 | 75.78 | 17.67 | 30.62 | 19.85 | 23.76 | - | - | - | - | 2.97 | Trigonal | --- |
| 10 | QMOF-2 | 1.76 | 18.44 | 5.50 | 13.12 | 18.50 | 45.70 | 18.44 | 18.50 | 13.12 | 6.47 | - | Hexagonal | --- |
| 11 | NaMn(HCOO)_3_ | 1.85 | 34.01 | 4.19 | 8.58 | - | - | - | - | - | - | - | cubic | --- |
| 12 | NH_4_Co(HCOO)_3_ | 1.87 | 48.60 | 24.21 | 19.44 | 36.66 | 76.36 | - | - | - | - | - | Hexagonal | --- |
| 13 | NH_4_Zn(HCOO)_3_ | 1.93 | 49.32 | 23.90 | 21.57 | 37.78 | 78.51 | - | - | - | 12.71- | - | Hexagonal | --- |
| 14 | GuaZn(HCOO)_3_ | 1.98 | 39.5 | 18.2 | 11.0 | 32.9 | 99.9 | 56.5 | 23.8 | 17.8 | 13.9 | - | Orthorhombic | --- |
| 15 | HyzZn(HCOO)_3_ | 2.00 | 46.3 | 32.6 | 14.4 | 18.9 | 60.8 | 68.7 | 25.4 | 11.0 | 21.7 | - | Orthorhombic | --- |
| 16 | MAMn(HCOO)_3_ | 1.80 | 97.1 | 11.6 | 14.5 | 59.3 | 103.4 | 151.5 | 16.1 | 58.5 | 6.2 | - | Orthorhombic | --- |
| 17 | MAZn(HCOO)_3_ | 1.93 | 37.6 | 19.4 | 5.9 | 28.0 | 64.0 | 70.7 | 23.3 | 22.8 | 7.4 | - | Orthorhombic | --- |
| 18 | DMAMg(HCOO)_3_ | 1.57 | 68.65 | 17.98 | 26.77 | 17.22 | 59.18 | 54.88 | 28.85 | 11.63 |  | - | Monoclinic | --- |
| 19 | DABCOH_2_K(ClO_4_)_3_ | 3.15 | 40.90 | 16.30 | 14.60 | - | - | - | - | - | - | - | cubic | 5 |
| 20 | Quartz | 2.65 | 86.80 | 6.83 | 58.23 | 12.02 | 105.83 | - | - | - | - | 18.11 | Trigonal | --- |
| 21 | BaTiO_3_ | 5.74 | 259 | 106 | 81 | 76 | 85 | - | - | - | 116 | - | Tetragonal | --- |
| 22 | Na_4_Al_3_(SiO_4_)_3_ | 2.08 | 59 | 12 | 33 | - | - | - | - | - | - | - | Cubic | --- |
| **Note**: ‘-‘ represents this parameter is nonexistent. ‘---‘ represents this work. | | | | | | | | | | | | | |  |

**Table S2.** Summary of the transverse wave velocities and longitudinal wave velocities (m/s) of the MOFs and inorganic materials.

| **No.** | **Materials** | **V_1max_** | **V_2max_** | **V_3max_** | **V_1min_** | **V_2min_** | **V_3min_** |
| --- | --- | --- | --- | --- | --- | --- | --- |
| 1 | MOF-5 | 3.13 | 3.67 | 6.84 | 1.67 | 1.67 | 5.70 |
| 2 | ZIF-8 | 1.03 | 1.09 | 3.11 | 0.91 | 0.91 | 3.03 |
| 3 | MAF-7 | 1.23 | 1.23 | 3.40 | 0.95 | 1.05 | 3.28 |
| 4 | DMOF-1loz | 2.57 | 3.26 | 5.90 | 0.34 | 0.61 | 2.97 |
| 5 | DMOF-1sq | 2.80 | 3.74 | 5.92 | 0.24 | 0.43 | 3.61 |
| 6 | MIL-47 | 4.29 | 7.08 | 9.94 | 0.56 | 2.83 | 5.60 |
| 7 | MIL-53(Al)lp | 4.39 | 5.47 | 8.86 | 0.51 | 2.36 | 5.02 |
| 8 | MIL-53(Ga)lp | 3.32 | 4.16 | 8.46 | 0.22 | 1.89 | 3.60 |
| 9 | QMOF-1 | 4.52 | 5.50 | 7.90 | 2.58 | 4.31 | 4.52 |
| 10 | QMOF-2 | 2.73 | 2.73 | 5.10 | 1.68 | 2.04 | 3.24 |
| 11 | NaMn(HCOO)_3_ | 2.63 | 2.84 | 4.29 | 2.15 | 2.15 | 3.72 |
| 12 | NH_4_Co(HCOO)_3_ | 3.22 | 3.22 | 6.39 | 2.53 | 2.70 | 5.10 |
| 13 | NH_4_Zn(HCOO)_3_ | 3.34 | 3.34 | 6.39 | 2.50 | 2.74 | 5.06 |
| 14 | GuaZn(HCOO)_3_ | 2.93 | 3.55 | 7.10 | 2.36 | 2.65 | 4.47 |
| 15 | HyzZn(HCOO)_3_ | 3.03 | 3.29 | 5.92 | 2.35 | 2.47 | 4.75 |
| 16 | MAMn(HCOO)_3_ | 5.03 | 6.03 | 9.17 | 1.86 | 2.84 | 6.26 |
| 17 | MAZn(HCOO)_3_ | 3.08 | 3.45 | 6.05 | 1.75 | 1.96 | 4.40 |
| 18 | DMAMg(HCOO)_3_ | 3.78 | 4.13 | 6.68 | 2.62 | 2.65 | 5.62 |
| 19 | DABCOH_2_K(ClO_4_)_3_ | 1.60 | 1.74 | 3.08 | 0.99 | 1.26 | 2.48 |
| 20 | Quartz | 4.69 | 4.79 | 6.67 | 3.59 | 4.22 | 5.64 |
| 21 | BaTiO_3_ | 3.76 | 4.50 | 7.21 | 2.39 | 3.66 | 3.85 |
| 22 | Na_4_Al_3_(SiO_4_)_3_ | 3.98 | 3.98 | 5.87 | 3.36 | 3.59 | 5.33 |

**Table S3.** Clamped-ion *ē* (C/m^2^), relaxed-ion *ê* (C/m^2^), and piezoelectric strain tensor *d* (pC/N) for QMOF-1.

| Index | *ē* | *ê* | *e* | *d* |
| --- | --- | --- | --- | --- |
| 11 | 0.011 | 0.220 | 0.231 | 4.60 |
| 14 | -0.015 | 0.728 | 0.713 | 23.64 |
| 15 | -0.036 | 0.608 | 0.572 | 17.54 |
| 22 | -0.042 | 0.286 | 0.244 | 2.90 |
| 31 | -0.074 | 0.204 | 0.130 | 0.90 |
| 33 | 0.038 | 0.052 | 0.09 | 2.38 |

**Table S4.** Summary of the BEC of QMOF-1 obtained from the DFPT calculation.

| Element | xx | yy | zz |
| --- | --- | --- | --- |
| Zn1 | 2.11 | 3.38 | 1.59 |
| Zn2 | 3.79 | 1.69 | 1.59 |
| Zn3 | 2.33 | 3.16 | 1.58 |
| Sum | 8.23 | 8.23 | 4.76 |

**Table S5.** Clamped-ion *ē* (C/m^2^), relaxed-ion *ê* (C/m^2^), and piezoelectric strain tensor *d* (pC/N) for QMOF-2.

| Index | *ē* | *ê* | *e* | *d* |
| --- | --- | --- | --- | --- |
| 14 | -0.010 | 0.043 | 0.033 | 2.52 |

**Table S6.** Clamped-ion *ē* (C/m^2^), relaxed-ion *ê* (C/m^2^), and piezoelectric strain tensor *d* (pC/N) for DMAMg(HCOO)_3_.

| Index | *ē* | *ê* | *e* | *d* |
| --- | --- | --- | --- | --- |
| 11 | -0.00027 | -0.10657 | -0.1068 | -1.2778 |
| 12 | -0.00687 | 0.07583 | 0.069 | 1.7277 |
| 13 | 0.03547 | -0.03681 | -0.0013 | -0.8705 |
| 15 | -0.0016 | -0.12701 | -0.1286 | -10.5367 |
| 24 | -0.0147 | -0.13543 | -0.1501 | -5.5998 |
| 26 | 0.00074 | 0.02985 | 0.0306 | 2.7909 |
| 31 | 0.01397 | -0.00752 | 0.0065 | 0.3552 |
| 32 | -0.00062 | -0.09817 | -0.0988 | -3.6703 |
| 33 | -0.02922 | 0.12238 | 0.0932 | 3.1870 |
| 35 | 0.03723 | -0.0588 | -0.0216 | -2.0416 |

**Figure S1** (a) 3D surfaces of the acoustic velocity for MOF-5. (b), (c) and (d) 2D plots of the acoustic velocity for MOF-5 projected normal to the (100), (010) and (001) planes, respectively. The green, red and blue lines represent *v*_1_, *v*_2_ and *v*_3_, respectively.


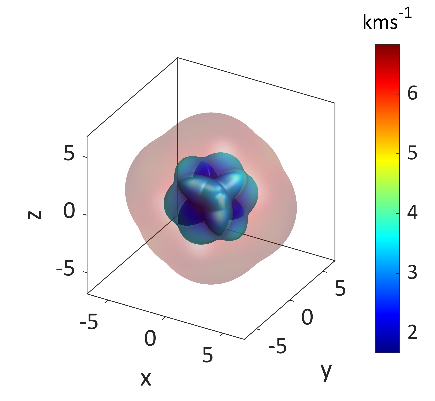

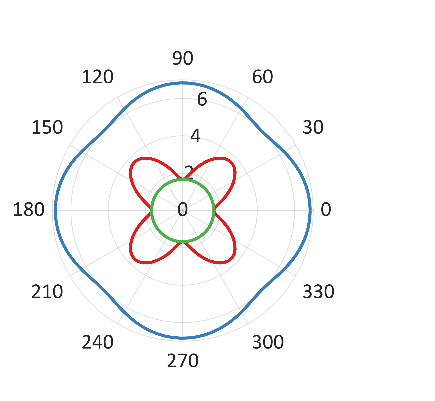

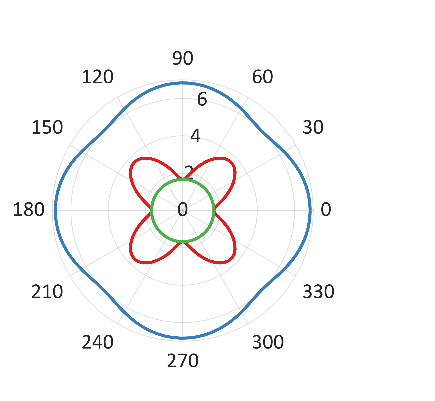

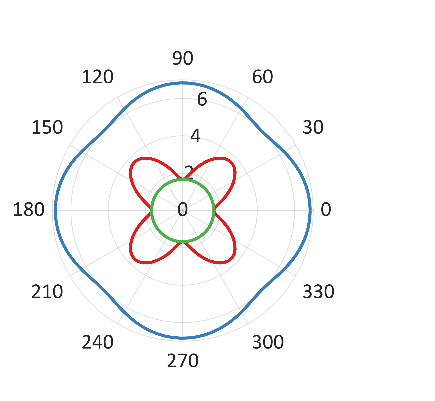


（a）

（b）

（c）

（d）


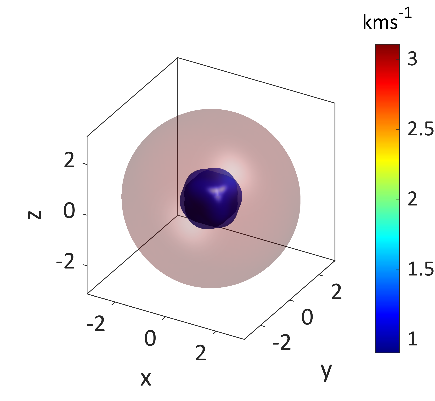

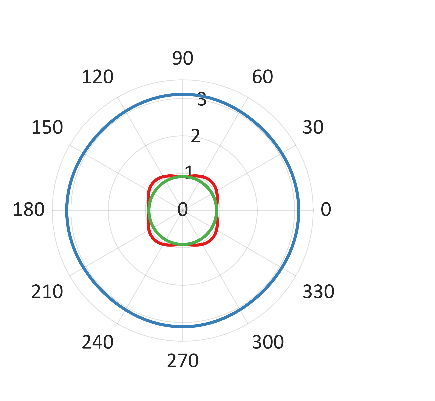

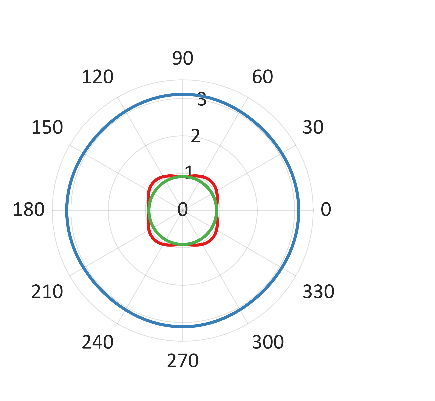

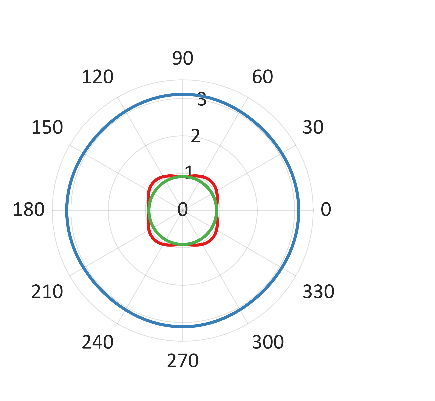


（a）

（b）

（c）

（d）

**Figure S2** (a) 3D surfaces of the acoustic velocity for ZIF-8. (b), (c) and (d) 2D plots of the acoustic velocity for ZIF-8 projected normal to the (100), (010) and (001) planes, respectively. The green, red and blue lines represent *v*_1_, *v*_2_ and *v*_3_, respectively.

**Figure S4** (a) 3D surfaces of the acoustic velocity for DMOF-1loz. (b), (c) and (d) 2D plots of the acoustic velocity for DMOF-1loz projected normal to the (100), (010) and (001) planes, respectively. The green, red and blue lines represent *v*_1_, *v*_2_ and *v*_3_, respectively.

**Figure S3** (a) 3D surfaces of the acoustic velocity for MAF-7. (b), (c) and (d) 2D plots of the acoustic velocity for MAF-7 projected normal to the (100), (010) and (001) planes, respectively. The green, red and blue lines represent *v*_1_, *v*_2_ and *v*_3_, respectively.


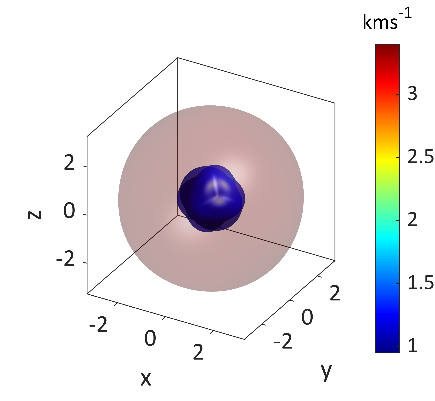

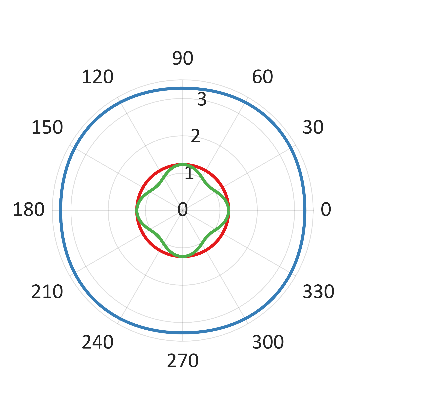

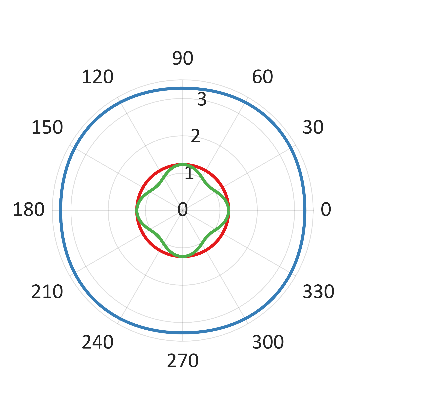

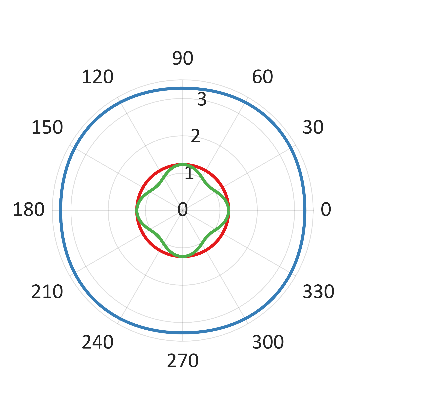


（a）

（b）

（c）

（d）


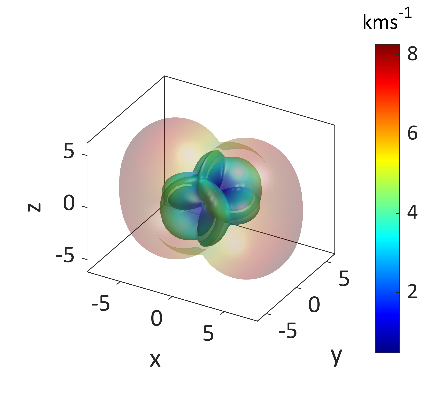

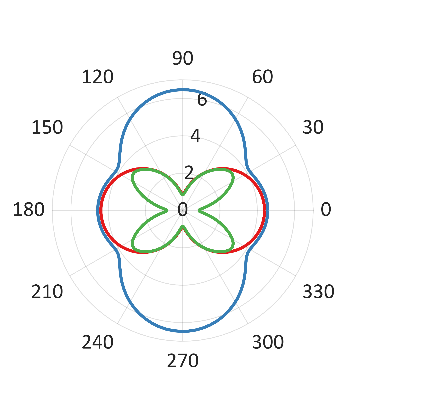

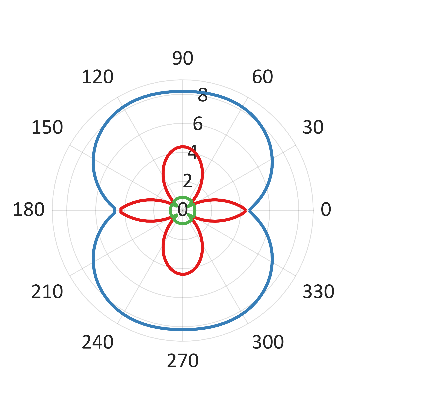

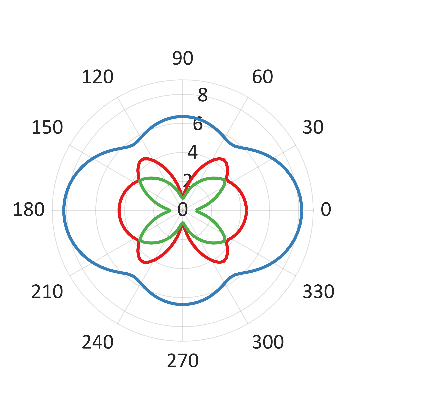


（a）

（b）

（c）

（d）

**Figure S6** (a) 3D surfaces of the acoustic velocity for MIL-47. (b), (c) and (d) 2D plots of the acoustic velocity for MIL-47 projected normal to the (100), (010) and (001) planes, respectively. The green, red and blue lines represent *v*_1_, *v*_2_ and *v*_3_, respectively.

**Figure S5** (a) 3D surfaces of the acoustic velocity for DMOF-1sq. (b), (c) and (d) 2D plots of the acoustic velocity for DMOF-1sq projected normal to the (100), (010) and (001) planes, respectively. The green, red and blue lines represent *v*_1_, *v*_2_ and *v*_3_, respectively.


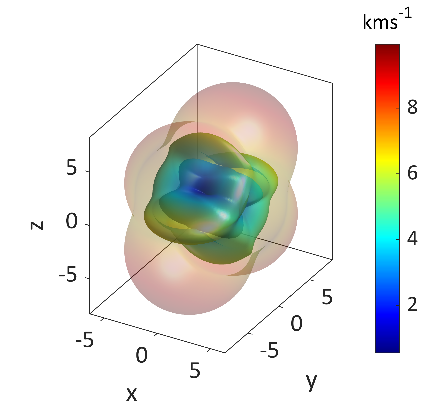

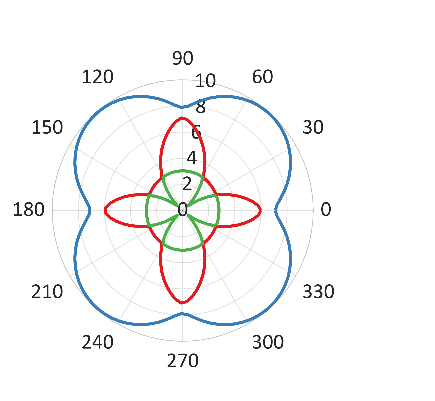

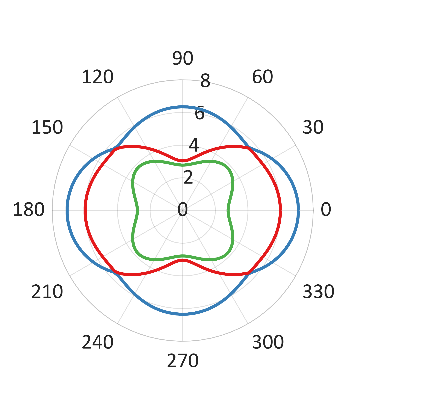

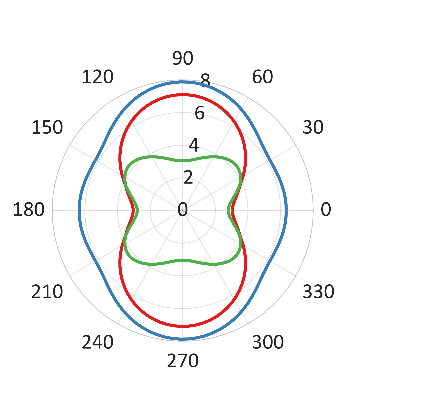


（a）

（b）

（c）

（d）


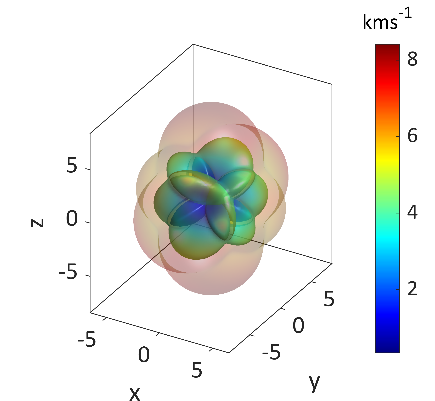

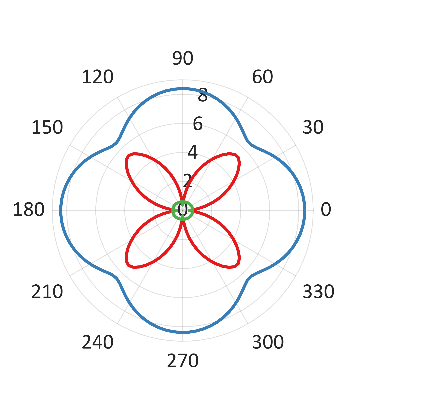

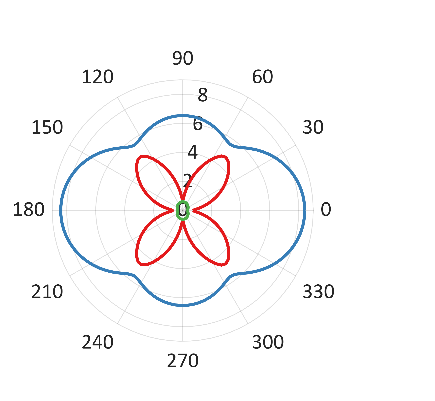

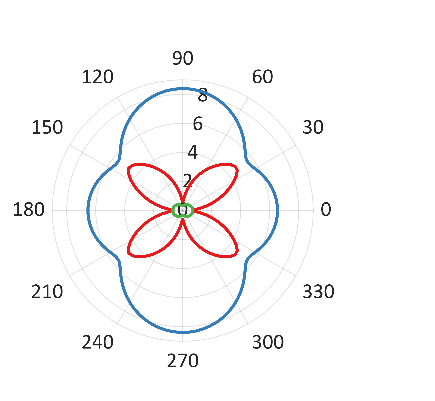


（a）

（b）

（c）

（d）

**Figure S8** (a) 3D surfaces of the acoustic velocity for QMOF-1. (b), (c) and (d) 2D plots of the acoustic velocity for QMOF-1 projected normal to the (100), (010) and (001) planes, respectively. The green, red and blue lines represent *v*_1_, *v*_2_ and *v*_3_, respectively.

**Figure S7** (a) 3D surfaces of the acoustic velocity for MIL-53(Al)lp. (b), (c) and (d) 2D plots of the acoustic velocity for MIL-53(Al)lp projected normal to the (100), (010) and (001) planes, respectively. The green, red and blue lines represent *v*_1_, *v*_2_ and *v*_3_, respectively.


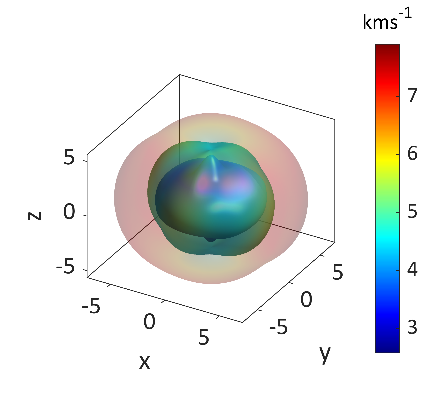

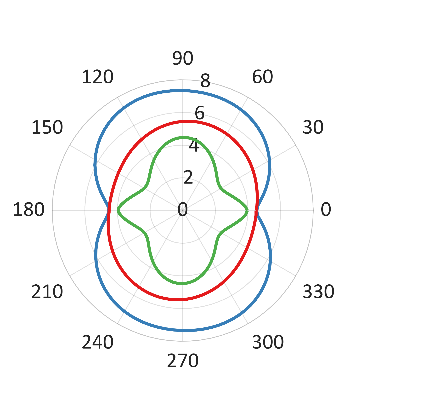

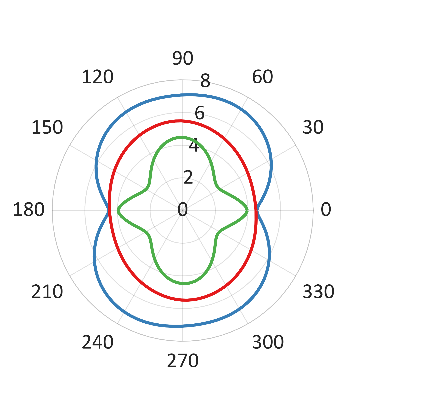

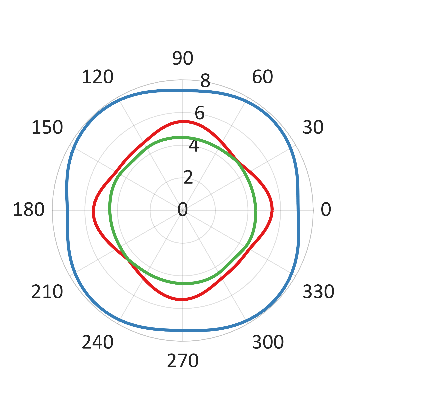


（a）

（b）

（c）

（d）


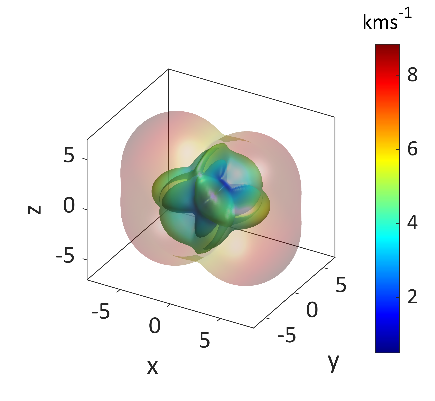

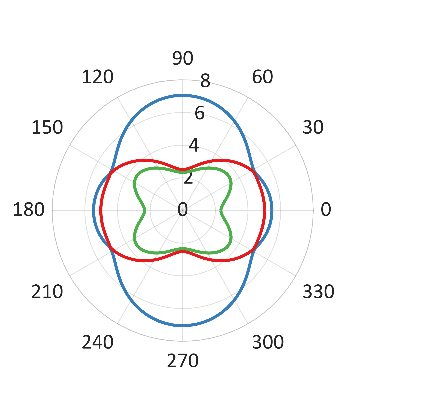

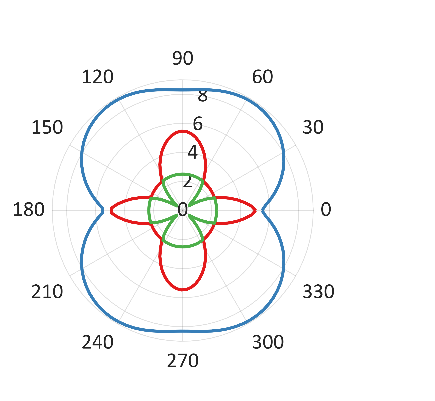

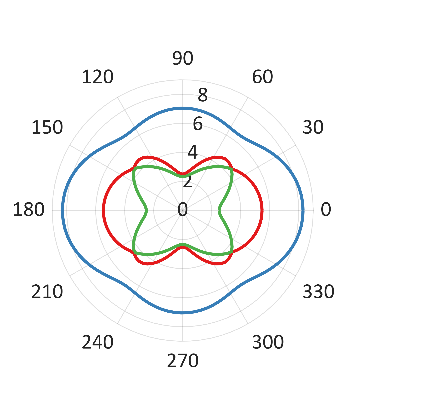


（a）

（b）

（c）

（d）


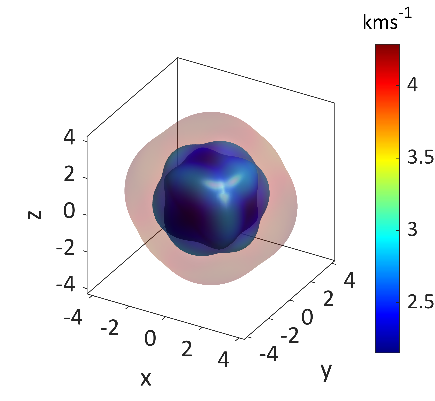

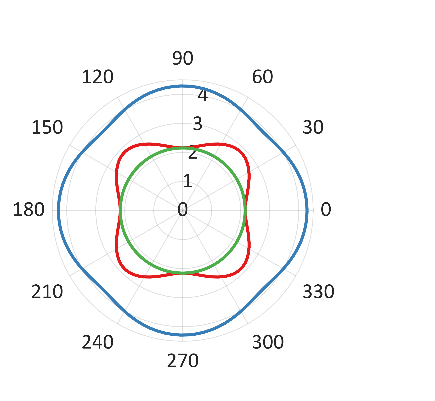

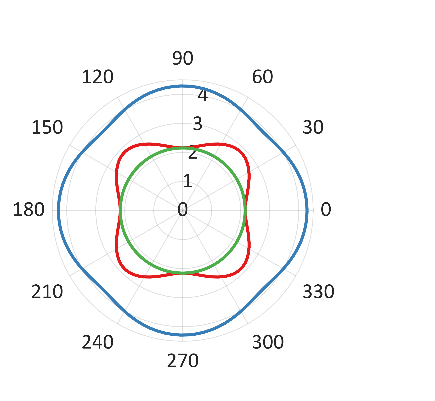

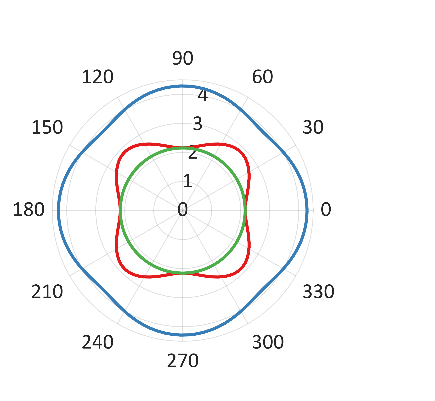


（a）

（b）

（c）

（d）

**Figure S10** (a) 3D surfaces of the acoustic velocity for NaMn(HCOO)_3_. (b), (c) and (d) 2D plots of the acoustic velocity for NaMn(HCOO)_3_ projected normal to the (100), (010) and (001) planes, respectively. The green, red and blue lines represent *v*_1_, *v*_2_ and *v*_3_, respectively.

**Figure S9** (a) 3D surfaces of the acoustic velocity for QMOF-2. (b), (c) and (d) 2D plots of the acoustic velocity for QMOF-2 projected normal to the (100), (010) and (001) planes, respectively. The green, red and blue lines represent *v*_1_, *v*_2_ and *v*_3_, respectively.


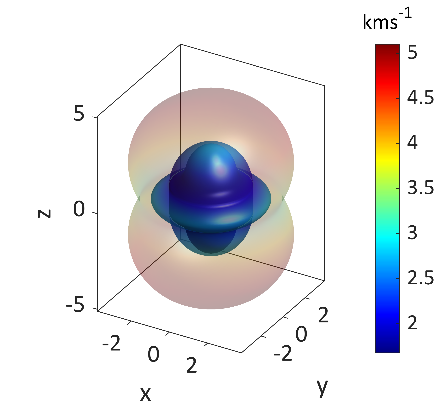

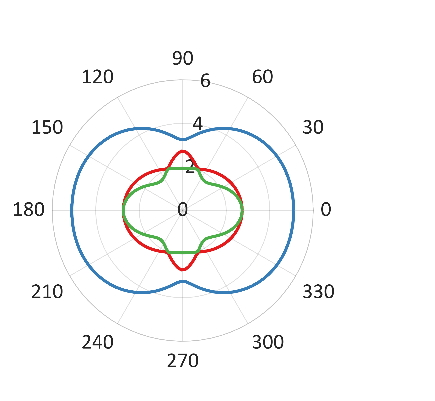

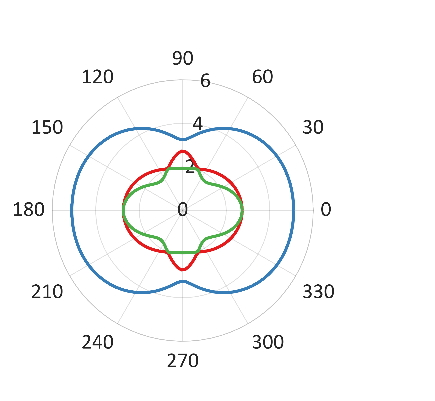

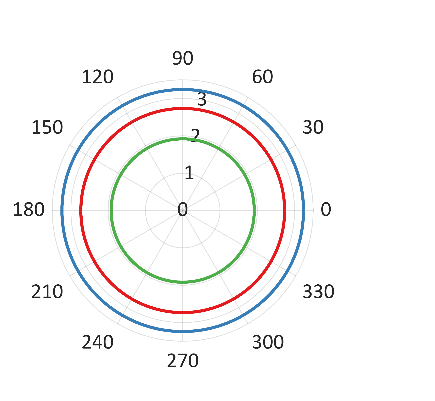


（a）

（b）

（c）

（d）


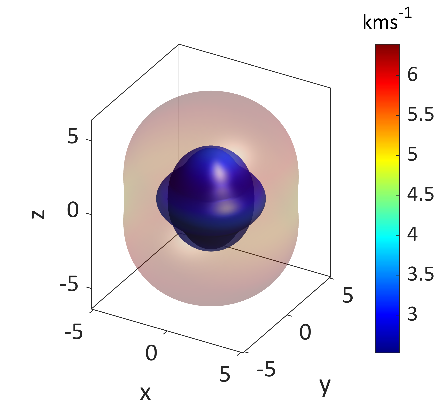

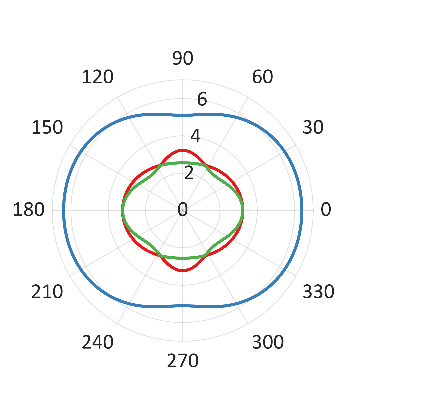

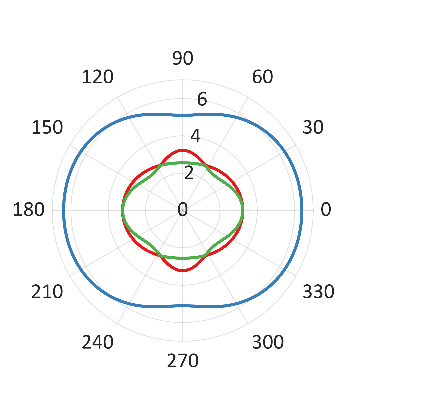

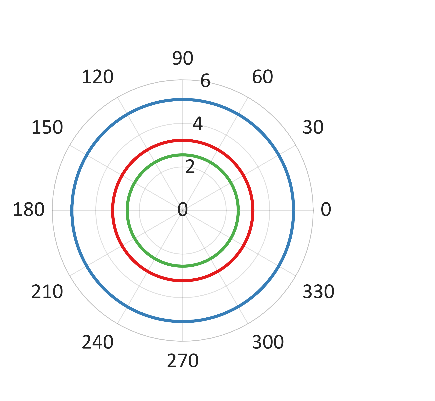


（a）

（b）

（c）

（d）

**Figure S12** (a) 3D surfaces of the acoustic velocity for NH_4_Zn(HCOO)_3_. (b), (c) and (d) 2D plots of the acoustic velocity for NH_4_Zn(HCOO)_3_ projected normal to the (100), (010) and (001) planes, respectively. The green, red and blue lines represent *v*_1_, *v*_2_ and *v*_3_, respectively.

**Figure S11** (a) 3D surfaces of the acoustic velocity for NH_4_Co(HCOO)_3_. (b), (c) and (d) 2D plots of the acoustic velocity for NH_4_Co(HCOO)_3_ projected normal to the (100), (010) and (001) planes, respectively. The green, red and blue lines represent *v*_1_, *v*_2_ and *v*_3_, respectively.


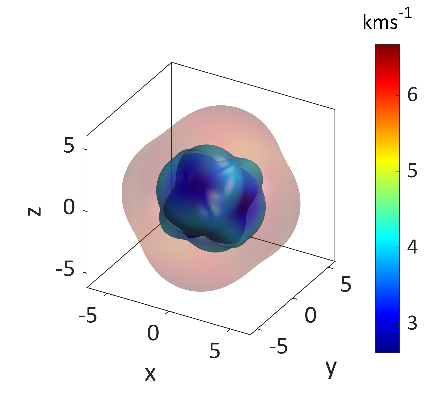

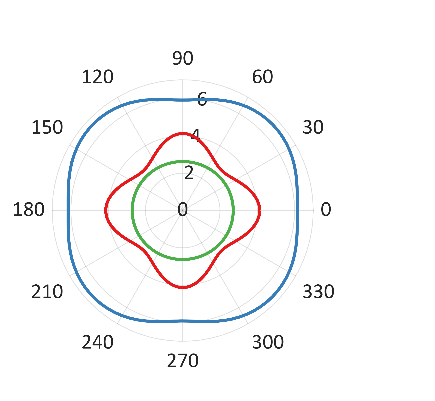

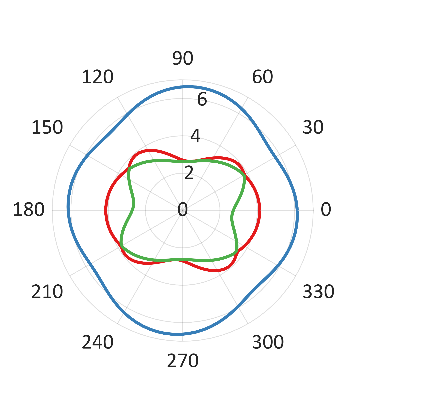

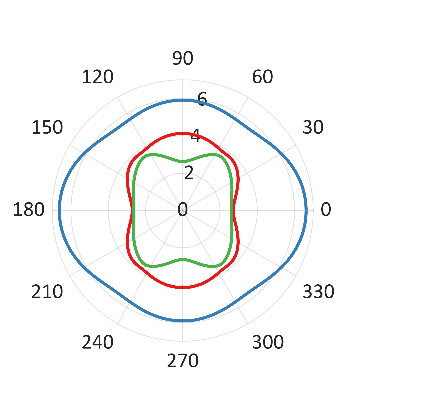


（a）

（b）

（c）

（d）


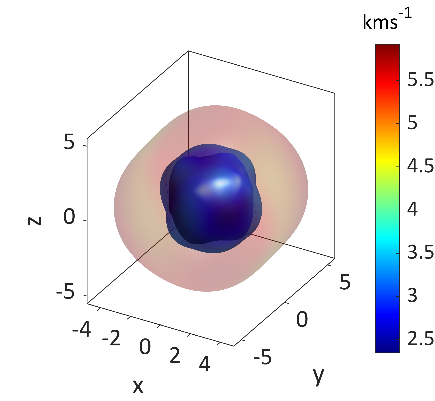

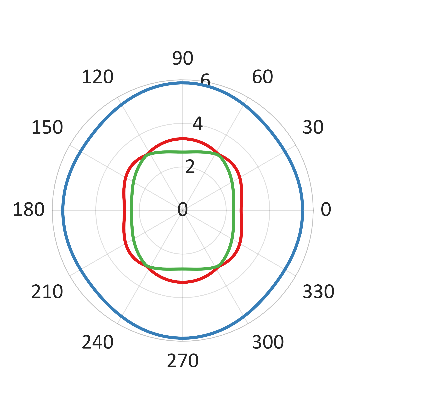

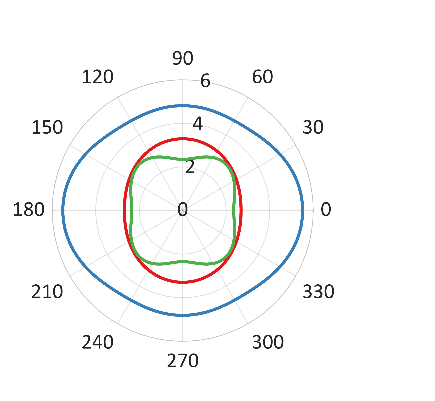

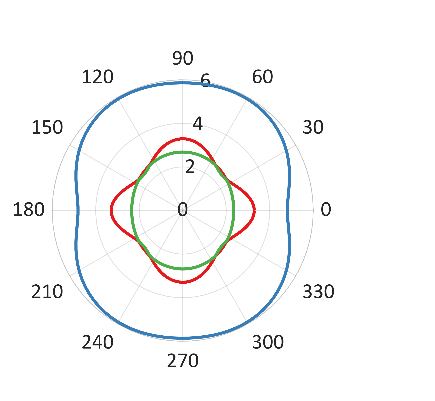


（a）

（b）

（c）

（d）

**Figure S14** (a) 3D surfaces of the acoustic velocity for HyzZn(HCOO)_3_. (b), (c) and (d) 2D plots of the acoustic velocity for HyzZn(HCOO)_3_ projected normal to the (100), (010) and (001) planes, respectively. The green, red and blue lines represent *v*_1_, *v*_2_ and *v*_3_, respectively.

**Figure S13** (a) 3D surfaces of the acoustic velocity for GuaZn(HCOO)_3_. (b), (c) and (d) 2D plots of the acoustic velocity for GuaZn(HCOO)_3_ projected normal to the (100), (010) and (001) planes, respectively. The green, red and blue lines represent *v*_1_, *v*_2_ and *v*_3_, respectively.


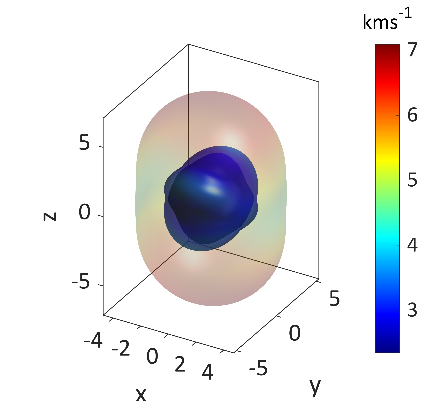

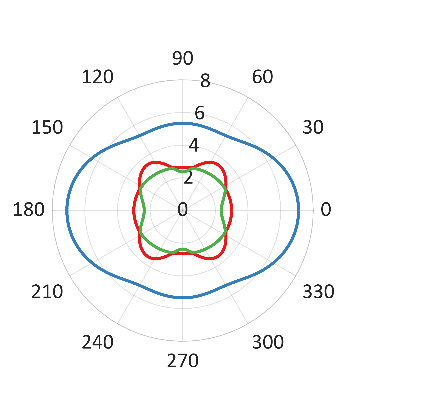

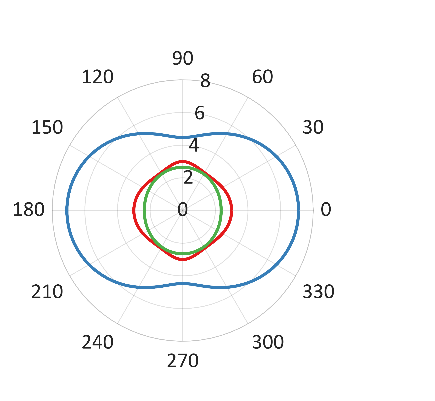

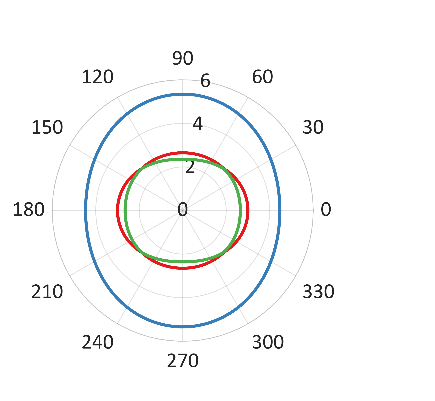


（a）

（b）

（c）

（d）


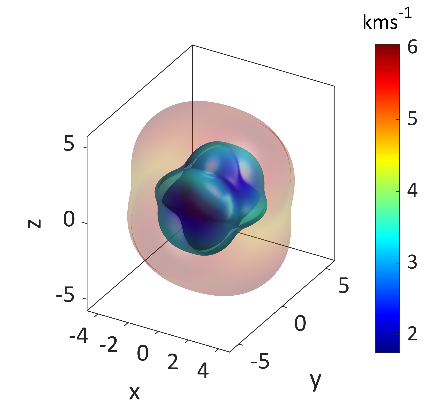

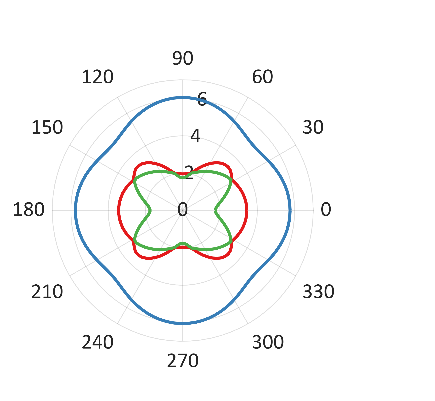

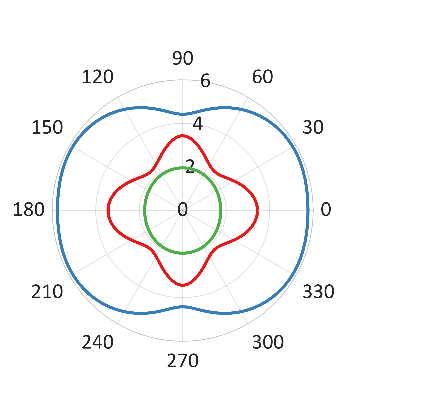

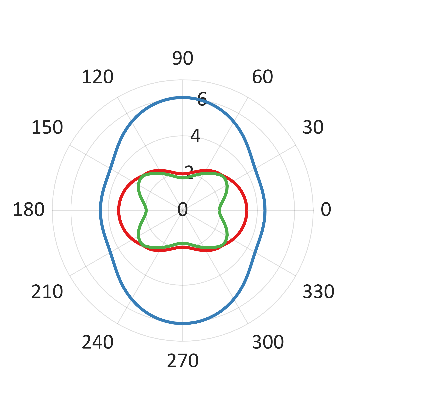


（a）

（b）

（c）

（d）


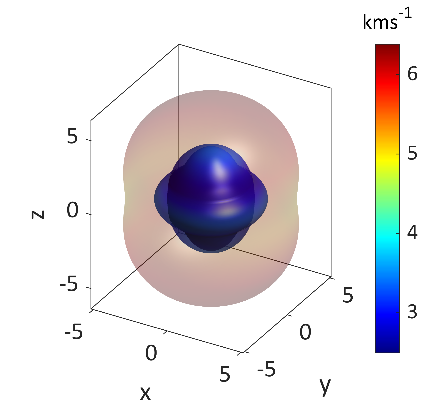

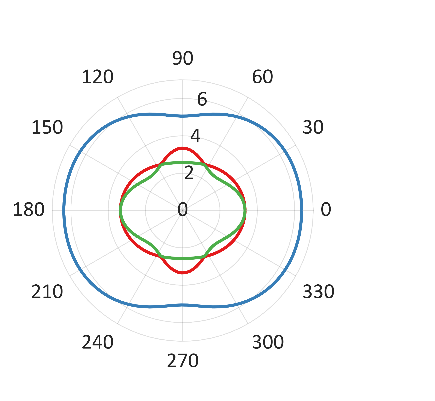

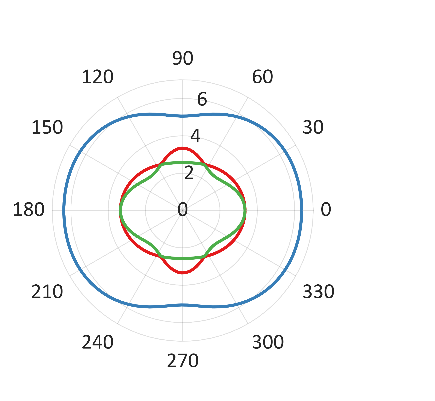

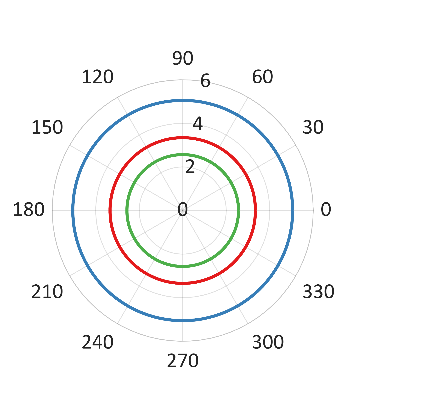


（a）

（b）

（c）

（d）

**Figure S16** (a) 3D surfaces of the acoustic velocity for MAZn(HCOO)_3_. (b), (c) and (d) 2D plots of the acoustic velocity for MAZn(HCOO)_3_ projected normal to the (100), (010) and (001) planes, respectively. The green, red and blue lines represent *v*_1_, *v*_2_ and *v*_3_, respectively.

**Figure S15** (a) 3D surfaces of the acoustic velocity for MAMn(HCOO)_3_. (b), (c) and (d) 2D plots of the acoustic velocity for MAMn(HCOO)_3_ projected normal to the (100), (010) and (001) planes, respectively. The green, red and blue lines represent *v*_1_, *v*_2_ and *v*_3_, respectively.

**Figure S18** (a) 3D surfaces of the acoustic velocity for DABCOH_2_K(ClO_4_)_3_. (b), (c) and (d) 2D plots of the acoustic velocity for DABCOH_2_K(ClO_4_)_3_ projected normal to the (100), (010) and (001) planes, respectively. The green, red and blue lines represent *v*_1_, *v*_2_ and *v*_3_, respectively.


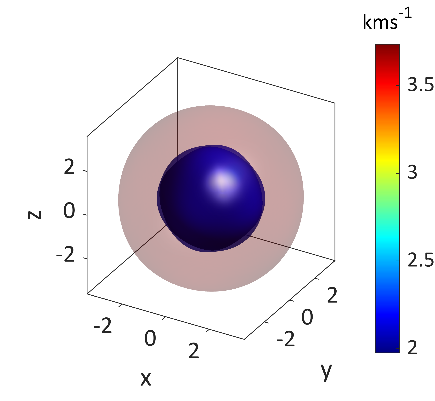

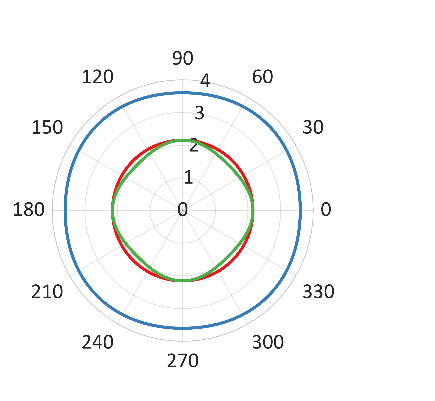

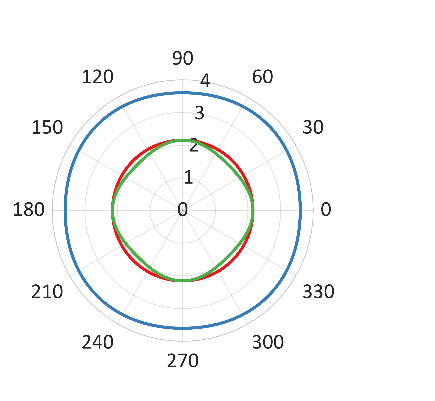

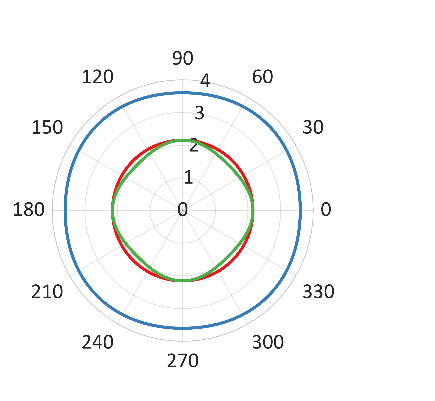


（a）

（b）

（c）

（d）

**Figure S17** (a) 3D surfaces of the acoustic velocity for DMAMg(HCOO)_3_. (b), (c) and (d) 2D plots of the acoustic velocity for DMAMg(HCOO)_3_ projected normal to the (100), (010) and (001) planes, respectively. The green, red and blue lines represent *v*_1_, *v*_2_ and *v*_3_, respectively.


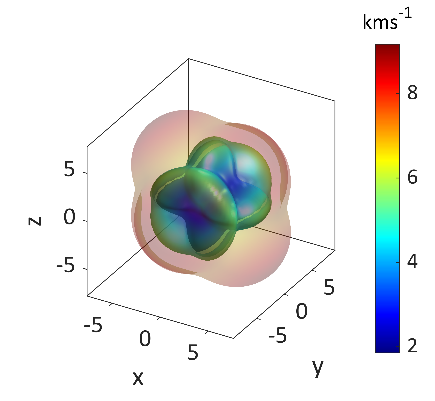

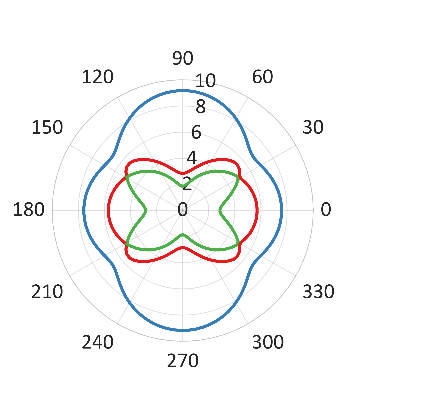

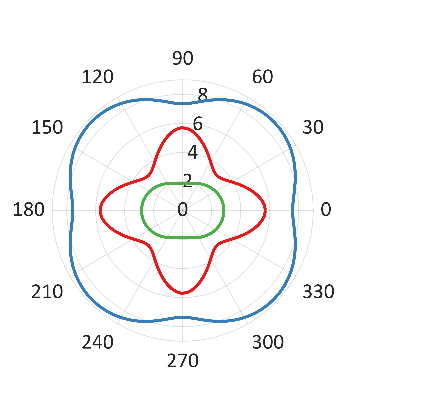

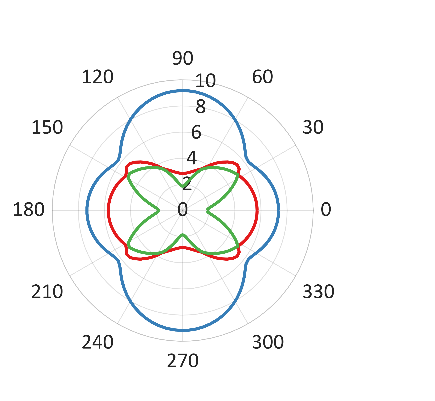


（a）

（b）

（c）

（d）


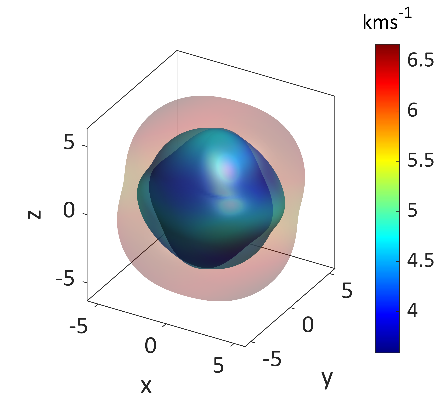

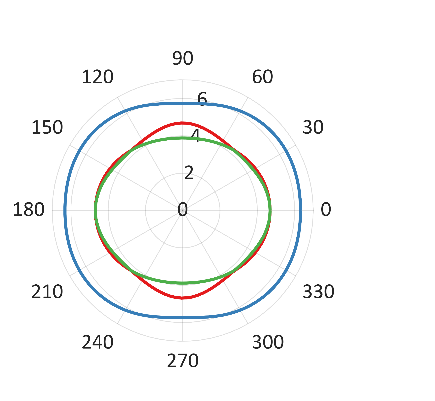

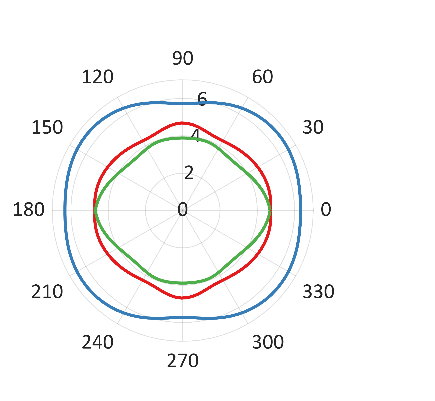

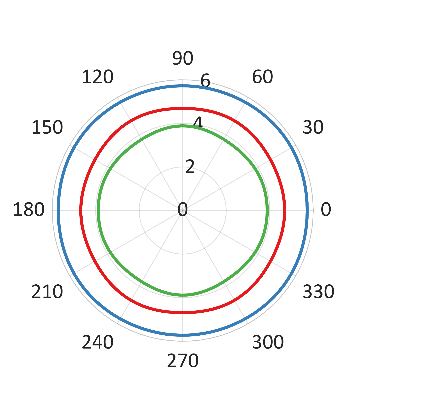


（a）

（b）

（c）

（d）


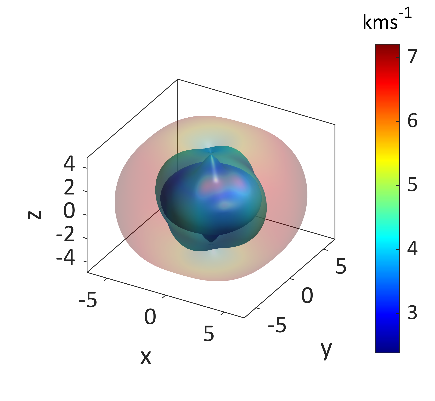

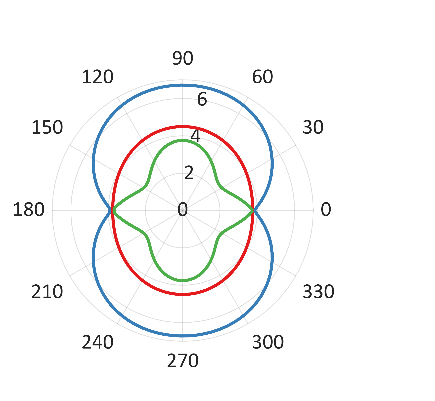

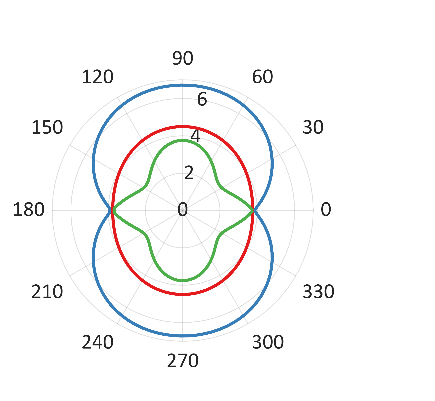

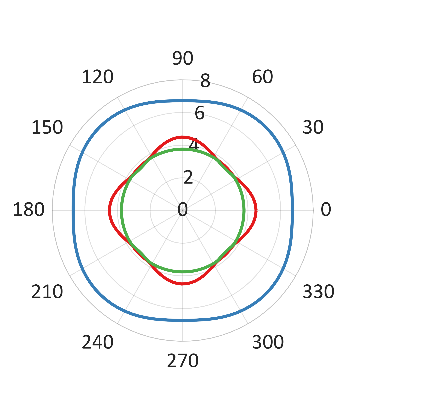


（a）

（b）

（c）

（d）

**Figure S20** (a) 3D surfaces of the acoustic velocity for BaTiO_3_. (b), (c) and (d) 2D plots of the acoustic velocity for BaTiO_3_ projected normal to the (100), (010) and (001) planes, respectively. The green, red and blue lines represent *v*_1_, *v*_2_ and *v*_3_, respectively.

**Figure S19** (a) 3D surfaces of the acoustic velocity for quartz. (b), (c) and (d) 2D plots of the acoustic velocity for quartz projected normal to the (100), (010) and (001) planes, respectively. The green, red and blue lines represent *v*_1_, *v*_2_ and *v*_3_, respectively.

**Figure S21** (a) 3D surfaces of the acoustic velocity for Na_4_Al_3_(SiO_4_)_3_. (b), (c) and (d) 2D plots of the acoustic velocity for Na_4_Al_3_(SiO_4_)_3_ projected normal to the (100), (010) and (001) planes, respectively. The green, red and blue lines represent *v*_1_, *v*_2_ and *v*_3_, respectively.


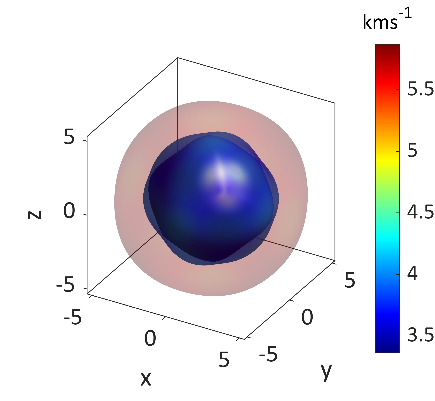

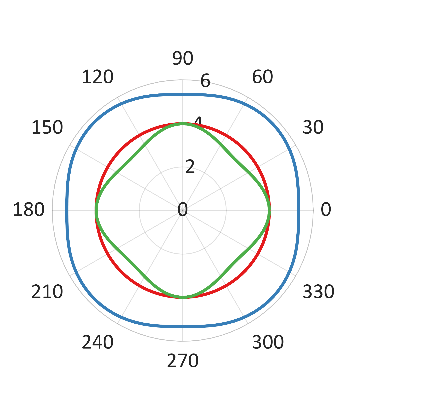

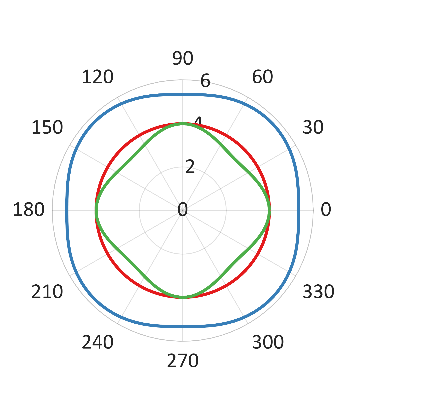

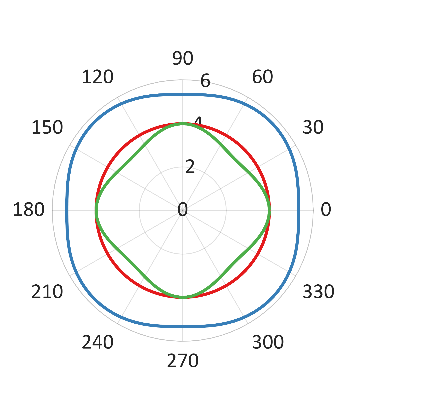


（a）

（b）

（c）

（d）


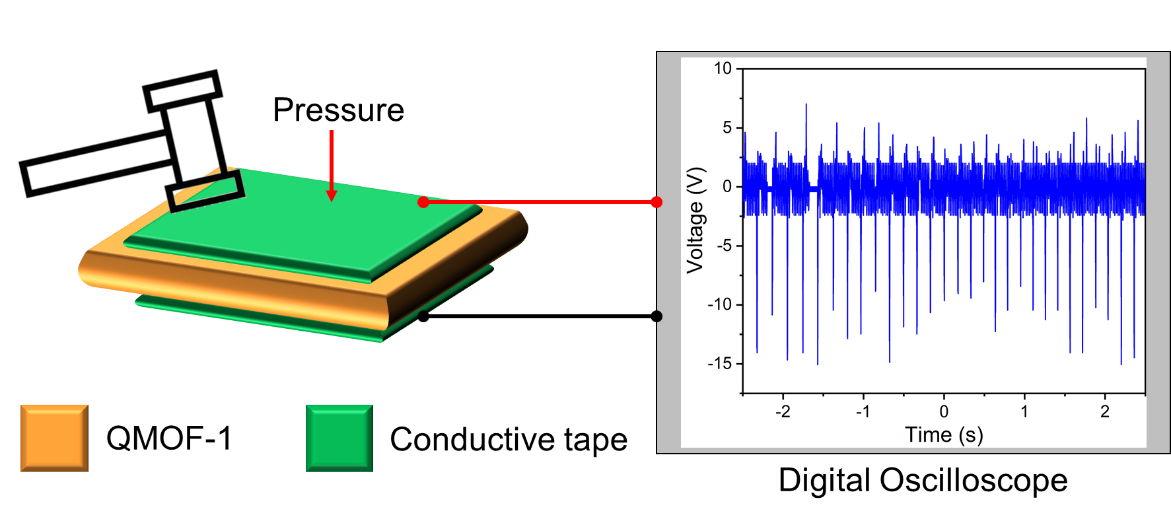


**Figure S22** (a) Schematic diagram of piezoelectric energy harvester based on polycrystalline pressed samples of QMOF-1. (b) Generated output voltages for QMOF-1 under certain mechanical press recorded by digital oscilloscope. The experiments were conducted based on a sandwich-structured Cu electrode-polycrystalline samples-Cu electrode device, under a periodic impact created by a mechanical contactor. The output voltage was measured using an oscilloscope (Keysight DSOX3024T).

**Reference**

[1] D. F. Bahr, J. A. Reid, W. M. Mook et al. “Mechanical properties of cubic zinc carboxylate IRMOF-1 metal-organic framework crystals,” *Physical Review B*, vol. 76, no. 18, article 184106, 2007.

[2] J. C. Tan, B. Civalleri, C. C. Lin et al., “Exceptionally low shear modulus in a prototypical imidazole-based metal-organic framework,” Physical Review Letters, vol. 108, no. 9, article 095502, 2012.

[3] H. Q. Gao, W. J. Wei, L. Y. Dong et al., “Enhanced framework rigidity of a zeolitic metal-azolate via ligand substitution,” *Crystals*, vol. 7, no. 4, article 7040099, 2017.

[4] A. U. Ortiz, A. Boutin, A. H. Fuchs et al., “Anisotropic elastic properties of flexible metal-organic frameworks: how soft are soft porous crystals?” *Physical Review Letters*, vol. 109, no. 19, article 195502, 2012.

[5] G. Q. Feng, X. X. Jiang, W. J. Wei et al., “High pressure behaviour and elastic properties of a dense inorganic-organic framework,” *Dalton Transactions*, vol. 45, no. 10, pp. 4303-4308, 2015.
